# Rosemary essenitial oil counters MnO_2_ nanoparticle-induced fertility deficits in rats via antioxidant mechanisms and upregulation of *StAR* signalling

**DOI:** 10.1038/s41598-025-06345-7

**Published:** 2025-06-20

**Authors:** Hager M. Ramadan, Nadia A. Taha, Ahmed M. Youssef, Asmaa S. Morsi

**Affiliations:** 1 Department of Physiology, Faculty of Veterinary Medicine, Cairo University, P.O. 12211, Giza, Egypt; 2 Packaging Materials Department, National Research Centre, P.O. 12622, Dokki, Giza, Egypt

**Keywords:** Rosemary essential oil, MnO_2_-nanoparticles, Male reproduction, Oxidative stress, Steroidogenesis, Gene expression, Physiology, Reproductive biology, Reproductive disorders

## Abstract

Manganese, an essential nutrient for male reproductive health, exerts dose-dependent effects, with excessive exposure—particularly to manganese dioxide nanoparticles (**MnO**_**2**_**-NPs**) from environmental or industrial sources inducing gonadal damage via oxidative stress, hormonal disruption, and impaired steroidogenesis. This study evaluated rosemary essential oil (**REO**) against **MnO**_**2**_**-NP**-induced reproductive dysfunction in male rats. Seventy-two Sprague–Dawley rats (130 ± 10 g) were divided into six groups (*n* = 12): Group I (deionized water), Group II (saline), Group III (**MnO**_**2**_**-NP**, 100 mg/kg bw/day), Group IV (**REO**, 250 mg/kg/day), Protective Group V (**REO** pre-treatment + **MnO**_**2**_**-NPs**), and Therapeutic Group VI (**MnO**_**2**_**-NPs + REO ** co-treatment) for 56 days. **MnO**_**2**_**-NP** exposure caused testicular injury, marked by elevated lipid peroxidation (↑malondialdehyde, ↑nitric oxide), suppressed antioxidants (↓total antioxidant capacity, ↓catalase, ↓glutathione), impaired sperm parameters (motility, count, morphology), and altered serum hormone levels (follicle-stimulating hormone, luteinizing hormone, testosterone). These effects correlated with downregulated steroidogenesis genes (***StAR***, **HSD-3β**,** CYP11A1**). Both Protective and Therapeutic REO treatment mitigated **MnO**_**2**_**-NPs** oxidative stress, restored hormonal balance, and normalized gene expression. Histopathology revealed reduced seminiferous tubule degeneration and enhanced spermatogenesis in **REO** groups. Findings demonstrate **REO’s** efficacy in alleviating **MnO**_**2**_**-NPs**induced reproductive toxicity via antioxidant and steroidogenic modulation, positioning **REO** as a promising therapeutic against nanomaterial-induced gonadotoxicity.

**Supplementary Information:**

The online version contains supplementary material available at 10.1038/s41598-025-06345-7.

## Introduction

The rapid integration of engineered nanoparticles into industrial and biomedical applications has unearthed a paradoxical challenge: their unique physicochemical properties, while enabling technological breakthroughs, also potentiate unforeseen biological hazards^1^. Among these, Manganese dioxide nanoparticles **MnO**_**2**_**-NPs**, employed in energy storage, catalysis, and environmental remediation, exemplify this duality, as emerging studies associate their bioaccumulation with male reproductive dysfunction—a pressing concern in industrialized regions^2,3^. **MnO**_**2**_**-NPs** instigate testicular injury via oxidative stress, hormonal disruption, and mitochondrial dysfunction, yet targeted interventions remain underdeveloped^4^. Although manganese serves as an essential enzymatic cofactor^5^, its nanoparticulate form circumvents physiological barriers, accumulating in gonadal tissues and inducing lipid peroxidation, glutathione depletion, and suppression of steroidogenic acute regulatory protein ***(StAR)***, a pivotal regulator of testosterone biosynthesis^6^. ***StAR*** mediates mitochondrial cholesterol transport, the rate-limiting step in steroidogenesis, regulated by luteinizing hormone **(LH)-**dependent **cAMP/PKA** signaling^7^. **LH** activates G protein-coupled receptors, elevating intracellular **cAMPs** to induce **CREB** phosphorylation. Phosphorylated **CREB** binds **cAMP response elements (CREs)** on the ***StAR*** promoter, synergizing with **steroidogenic factor 1 (SF-1)** to enhance transcription. Upregulated ***StAR*** promotes cholesterol transfer to **CYP11A1 (P450scc)** in the mitochondrial inner membrane, catalyzing pregnenolone synthesis—the precursor for gonadal steroid hormones. Post-translational phosphorylation further modulates ***StAR*** activity, optimizing steroidogenic output^8^. **MnO**_**2**_**-NPs** disrupt this pathway, downregulating ***StAR***, **CYP11A1**, and **HSD-3β**, thereby impairing spermatogenesis and steroidogenic capacity^9^.

Rosemary essential oil **(REO)** (*Rosmarinus officinalis* L.), a phytochemical complex enriched with carnosic acid, rosmarinic acid, camphor, and polyphenols (e.g., flavonoids, isoflavones), enhances fertility through dual antioxidative and endocrine-modulatory mechanisms^10,11^. Its phenolic constituents scavenge reactive oxygen species **(ROS)**, attenuating oxidative stress in steroidogenic tissues such as Leydig cells. This action preserves mitochondrial membrane integrity, inhibits lipid peroxidation, and boosts endogenous antioxidant enzyme activity, thereby safeguarding spermatogenic cells and preventing sperm quality deterioration^12^. Concurrently, **REO** modulates endocrine pathways by reactivating ***LH/CREB/StAR*** signalling, restoring testosterone biosynthesis via upregulation of ***StAR*** gene expression in testicular tissues. **REO**’s phenolic constituents enhance hormonal regulation by inhibiting **ROS**-mediated suppression of steroidogenic enzymes, thereby restoring testosterone biosynthesis^13^. The synergistic interplay between polyphenol-mediated anti-inflammatory effects and endocrine pathway modulation enhances reproductive function, notably improving sperm motility and viability^14^.

This study fills a critical gap in managing nanoparticle-induced testicular damage by introducing rosemary essential oil **(REO)** as the first natural treatment that simultaneously tackles oxidative stress and hormonal dysfunction. **REO** uniquely combines **ROS** neutralization (via α-pinene, carnosic acid) with direct restoration of testosterone production by reactivating ***LH/StAR*** signaling and steroidogenic enzymes **(HSD-3β**,** CYP11A1).** This dual-action mechanism—unreported in prior studies—offers a transformative solution for holistic recovery of reproductive health, bridging the divide between antioxidant therapies and endocrine restoration.

## Materials and methods

### Animals and experimental design

Seventy-two male *Sprague-dawley* rats (130 ± 10 g) and aged 10–12 weeks were purchased from Vacsera animal house colony, Cairo, Egypt. All experimental procedures were carried out in accordance with the NIH Guide for the Care and Use of Laboratory Animals, the ARRIVE guidelines for in vivo experiment reporting, and received approval from the Institutional Animal Care and Use Committee **(VET.CU. IACUC)** at the Faculty of Veterinary Medicine, Cairo University, following the Animal Use Protocol **(Vet CU 25122023884).** Water was given to rats ad-libitum while ration was given to rats according to National Research Council^15^. The rats were fed on standard granulated ration (2.5% Crude Fat, 23% Crude Protein, 5.5% Crude Fibers and Metabolizable Energy = 3600 Kcal/Kg). Following a two-week acclimation period, 72 rats were randomly allocated into six experimental groups (*n* = 12 per group), each housed in three replicate cages (four rats per cage) to minimize crowding-related stress. To account for route-specific administration effects, two vehicle control groups were utilized: an oral saline vehicle and a subcutaneous saline vehicle. The study duration spanned 56 days, encompassing the full spermatogenic cycle of the species to enable systematic assessment of germ cell maturation across all developmental stages.

### Experimental groups

1. Oral Control Group (CO): Received 1 mL/kg body weight (bwt) sterile saline via daily oral gavage.
2. Subcutaneous Control Group (CS): Administered 1 mL/kg bwt sterile saline via daily subcutaneous injection [vehicle for the manganese oxide nanoparticles (**MnO**_**2**_**-NPs**)]
3. **MnO**_**2**_**-NP** Exposure roup (III): Rats received a daily subcutaneous injection of manganese oxide nanoparticles (**MnO**_**2**_**-NPs**; 100 mg/kg bwt) dissolved in saline, based on prior toxicity studies^2^.
4. REO Alone Group (IV): Rats were orally administered rosemary essential oil (REO; 250 mg/kg bwt), a dosage established for bioactive efficacy^10^.
5. Protective Group (V): Rats received **REO** (250 mg/kg bwt, oral) 30 min prior to **MnO**_**2**_**-NP** (100 mg/kg bwt, subcutaneous) administration.
6. Therapeutic Group (VI): Rats were administered **MnO**_**2**_**-NP** (100 mg/kg bwt, subcutaneous) followed by **REO** (250 mg/kg bwt, oral) after a 30-minute interval.

### Chemicals

#### Preparation and characterization of manganese oxide nanoparticles (MnO_2_-NPs)

**Fig. 1:**
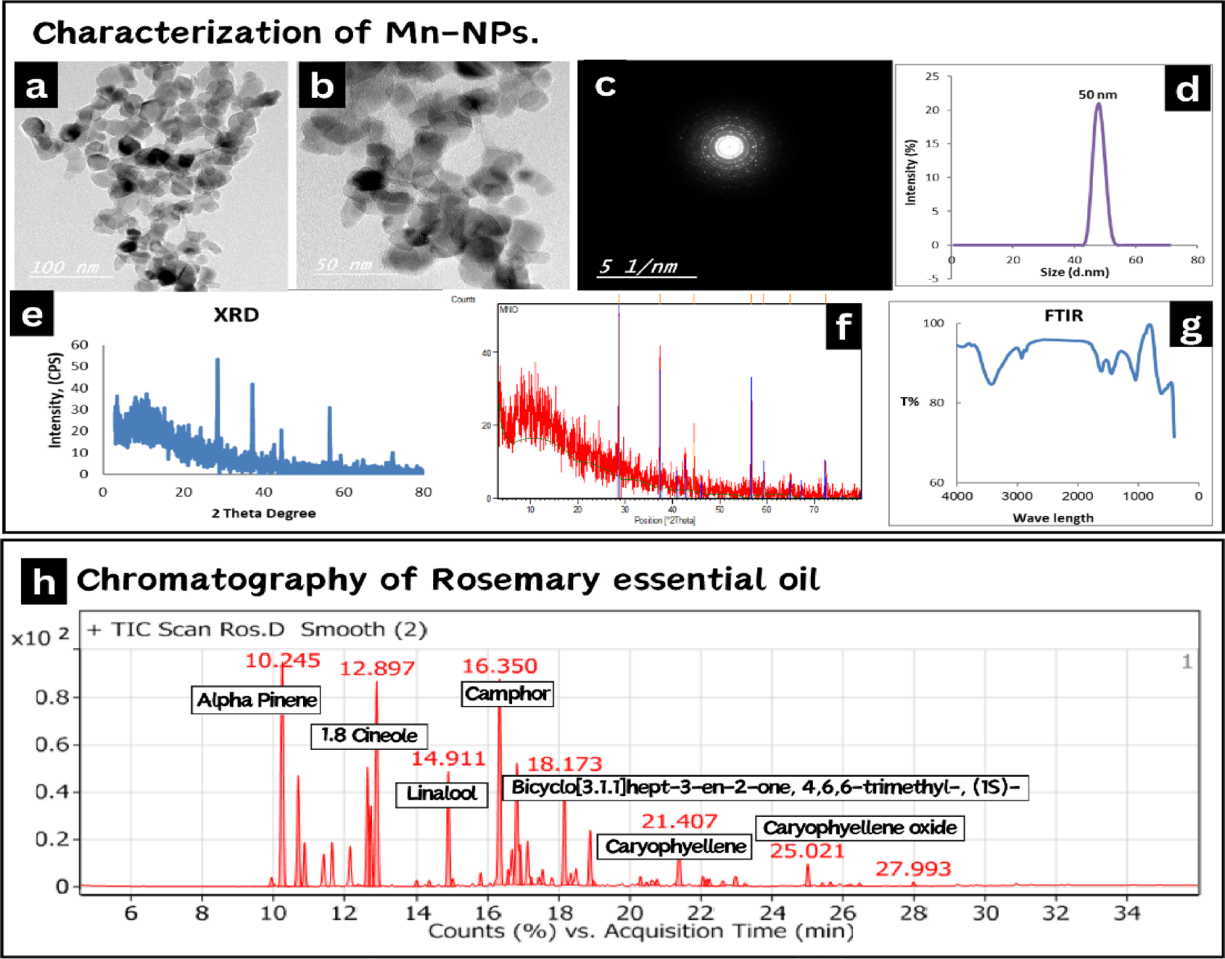
Characterization of MnO_2_-NPs . Including (**a–c**) High-resolution transmission electron microscope image of MnO_2_-NPs exhibited irregular shape, with an average size of 50–100 nm. (**d**) Dynamic light scattering analysis showed particle size 50 nm. (**e**,**f**) X-ray powder diffraction patterns of MnO_2_- NPs. (**g**) Fourier transforms infrared spectroscopy of MnO_2_-NPs. Gas chromatography–mass spectrometry analysis (GC-MS) of rosemary essential oil. (**h**) Shows the fragmentation pattern of its components.

Manganese oxide nanoparticles (**MnO**_**2**_**-NPs**) were prepared by the green synthesis method according to^16^. The preparation and characterization of **MnO**_**2**_**-NPs** were performed at the central laboratories network, National Research Centre, Cairo, Egypt. A High-Resolution Transmission Electron Microscope (HR-TEM) with an accelerating voltage of 200 kV was used to image the morphology of NPs (Tecnai G2, FEI, Netherlands). A TEM micrograph (Fig. 1a–c) reveals that **MnO**_**2**_**-NPs** have lattice fringes shape, which points to the development of a good nanocrystalline structure. The particles’ size ranges from 50 to 100 nm. A Nano-zeta sizer (Malvern, ZS Nano, U.K.) was used to assess the particle size using dynamic light scattering (DLS). The findings of the particle size analysis investigation (Fig. 1d) showed that the produced MnO_2_-NPs have a size range of 50 nm. The findings demonstrate the creation of MnO2 nanomaterials. The phase and structure of the produced NPs were investigated using an X-ray diffractometer (XRD, X’Pert Pro, Malvern Analytical Ltd, Malvern, UK) at 40 kV and 30 mA, the standard ICCD was used to evaluate the data. The corresponding results are shown in (Fig. 1e, f). The sample’s various diffraction peaks correlate to 2θ values of 18.03, 28.71, 37.58, 47.78 and 59.01, which match the presence of **MnO**_**2**_**-NPs** with lack of impurity phases. Fourier transforms infrared spectroscopy (FTIR) is carried out and these considerations are considered: 16 scans, spectral range 0–4500 cm^− 1^, spectral resolution 4 cm^− 1^. The software used to utilize FTIR was Opus (Bruker, Germany), VERTEX 70, RAMII. Multiple absorption bands at 762, 570, and 435 cm^− 1^ are visible in the FT-IR spectra of **MnO**_**2**_**-NPs**, as shown in (Fig. 1g). The metal oxide nanoparticles, such as **MnO**_**2**_ **NPs**, typically exhibit an absorption peak in the fingerprint area below 1000 nm wavelength which is due to inter-atomic vibrations. The **Mn–O** bond can be identified by the absorption bands at 570 and 530 cm^− 1^. The peaks at 1690 and 1494 cm^− 1^ are associated with manganese atoms and -O-H bending vibrations.

### Gas chromatography–mass spectrometry analysis (GC-MS) of Rosemary essential oils (REO)

The essential oil of *Salvia rosmarinus* Spenn. (syn. *Rosmarinus officinalis* L.; family Lamiaceae) was procured from the Essential Oils Extraction Unit, National Research Centre (**NRC**,** Egypt; Lot No. 19-02-2024**). Chemical profiling was performed via gas chromatography-mass spectrometry (**GC-MS**) at the NRC Central Laboratories Network. Constituents were identified by comparing mass spectral fragmentation patterns against the Wiley 11th Edition and NIST 2020 databases, with match similarity indices ≥ 90%. Results are reported in Table 1; Fig. 1h.

**Table 1:** Main constituents of REO.

Constituents	%
Alpha-Pinene	13.63
Camphene	5.8
Beta-Pinene	1.64
Beta-Myrcene	1.96
1-Octen-3-ol	2.17
α-Terpinene	0.18
D-Limonene	5.77
o-Cymene	3.87
1.8 Cineole	11.51
Borneol	6.28
Borneol acetate	2.69
5,7,8-trimethyltocol	0.29
Camphor	11.72
Benzene, 1-methyl-4-(1-methylethenyl)	0.28
Filifolone	0.37
Cyclohexanol, 2-methyl-5-(1-methylethenyl)-	0.93
Copaene	0.41
Isopulegol acetate	0.15
1-Isopropenyl-3-propenylcyclopentane	0.4
Eugenol	0.3
Caryophyllene	2.72
Humulene	0.49
Gamma.-Muurolene	0.4
Aromandendrene	0.35
Delta.-Cadinene	0.66
Trans-Calamenene	0.16
Caryophyllene oxide	1.09
γ-Eudesmol	0.14
Tau-Cadinol	0.22
14-Hydroxycaryophyllene	0.15

### Blood collection

At the end of the experiment, blood samples were collected between 8:00 and 10:00 a.m. to mitigate circadian rhythm in hormones levels. Blood was drawn via retro-orbital sinus puncture under anesthesia induced with isoflurane (30% in propylene glycol (Sigma-Aldrich, St. Louis, MO, USA; Catalog #I4381), following established protocols^17^. Post-collection, samples were processed without anticoagulant for serum samples, and they were centrifuged for 15 min at 4000 rpm. Sera were stored at -20 °C to be used for further examination.

### Tissue collection

The animals were sacrificed after anesthesia overdosed by cervical dislocation and testicular tissues and epididymis were collected. Following semen collection via epididymal dissection for comprehensive analysis, testes tissues were surgically excised from all rats. Tissues were rinsed in physiological saline (0.9% NaCl) and partitioned into two cohorts: *Snap-frozen cohort*: Tissues were immediately snap-frozen in liquid nitrogen and stored at − 80 °C for subsequent RNA/protein extraction, oxidative stress marker, and gene expression profiling. *Fixed cohort*: Tissues were immersion-fixed in 10% neutral buffered formalin (24–48 h) for histomorphology evaluations, followed by paraffin embedding and hematoxylin-eosin staining.

### Measured parameters

#### Body weights and relative testes weight

The final body weight and testicular weight in grams of all rats were recorded at the end of the experiment. Additionally, relative testes weight of rats of all groups were calculated using the following equation as described by^18^.

$$\:\text{R}\text{e}\text{l}\text{a}\text{t}\text{i}\text{v}\text{e}\:\text{t}\text{e}\text{s}\text{t}\text{i}\text{c}\text{u}\text{l}\text{a}\text{r}\:\text{w}\text{e}\text{i}\text{g}\text{h}\text{t}=\:\left(\frac{\text{T}\text{e}\text{s}\text{t}\text{i}\text{c}\text{u}\text{l}\text{a}\text{r}\:\:\text{w}\text{e}\text{i}\text{g}\text{h}\text{t}\:\text{i}\text{n}\:\text{g}\text{r}\text{a}\text{m}\text{s}\:}{\text{B}\text{o}\text{d}\text{y}\:\text{w}\text{e}\text{i}\text{g}\text{h}\text{t}\:\text{i}\text{n}\:\text{g}\text{r}\text{a}\text{m}\text{s}}\right)\:\times\:\:100$$

#### Semen analysis

Evaluation of epididymal sperm count, motility, viability and morphological abnormalities were done according to methods described by^19^.

### Oxidative stress indices

Tissue preparation, tissue samples were homogenized using an Ultra-Turrax T-25 homogenizer in 5–10 ml of cold PBS (phosphate-buffered saline) solution at pH 7.4 per 1 g of tissue. Centrifuge at 4000 rpm for 15 min, then collect the supernatant for further examination^20^.

Evaluation of redox status, serum total antioxidant capacity (TAC) and testicular antioxidant parameters including catalase (CAT) activity, reduced glutathione (GSH) concentration, malondialdehyde (MDA) concentration and nitric oxide (NOx) were detected spectrophotometrically according to the method described by^21–25^ respectively.

### Determination of serum reproductive hormones

Serum free testosterone was detected according to the method of^26^, using commercial kits purchased from Bios Company, USA (CAT. No10007). Serum follicle stimulating hormone (FSH) and luteinizing hormone (LH) were measured according to the method of^27^ using commercial kits purchased from ELK Company, China, (CATNO. ELK1315 and ELK2367, respectively).

### Determination of *StAR* signaling and its associated genes (HSD-3β and CYP11A1) in testicular tissue

Isolation of Total RNA, total RNA was isolated from the samples using the GeneJET RNA Purification Kit (Thermo Scientific, catalog #K0731/K0732). The extraction protocol included cell lysis, removal of genomic DNA contamination, and column-based RNA purification. RNA quality was evaluated by assessing purity and concentration with a NanoDrop™ One spectrophotometer (Thermo Fisher Scientific, USA), where A260/A280 ratios between 1.8 and 2.0 confirmed acceptable purity. RNA integrity was further validated via 1% agarose gel electrophoresis (100 V, 30 min), which resolved distinct 28 S and 18 S ribosomal RNA bands.

cDNA Synthesis from Total RNA, the Enzynomics cDNA Synthesis Kit (#RT220, RT221) was used to synthesize complementary DNA (cDNA). The reverse transcription reaction was performed in a thermocycler under optimized conditions to ensure accurate and efficient conversion of RNA into cDNA. This process utilized a combination of reverse transcriptase enzyme, primers, and dNTPs to generate high-quality cDNA for downstream applications.

qPCR-based Gene Expression Analysis, the synthesized cDNA was used as a template for quantitative polymerase chain reaction (qPCR). Quantitative PCR amplification was performed using Maxima SYBR Green qPCR Master Mix (Thermo Scientific, Waltham, MA, USA; Cat# K0251) on a Q3 Tower Real-Time PCR System (Analytik Jena, Jena, Germany). Reactions (20 µL) contained 2 µL cDNA template and gene-specific primers (Table 2) for ***CYP11A1***, ***StAR***, and **HSD-3β**. Primers were designed using Primer3 (v0.4.0; http://bioinfo.ut.ee/primer3-0.4.0/)^28^. Cycling conditions: 95 °C for 3 min; 40 cycles of 95 °C for 15 s, 60 °C for 30 s. Amplification specificity was confirmed by melting curve analysis (65–95 °C). ***β-actin*** served as internal control, with expression stability validated across experimental groups using NormFinder software^29^.

Gene Expression Relative Quantification (RQ), the ΔΔCt method, as described by^30^, was applied to determine relative gene expression levels. This approach involved normalizing the cycle threshold (Ct) values of target genes to those of an internal reference gene, followed by comparison with a control group. The fold-change in gene expression was determined using the formula:

$$\:\varDelta\:\text{C}\text{t}\:=\text{C}\text{t}\:\left(\text{t}\text{a}\text{r}\text{g}\text{e}\text{t}\text{e}\text{d}\:\text{g}\text{e}\text{n}\text{e}\right)\--\:\text{C}\text{t}\:\left(\text{h}\text{o}\text{u}\text{s}\text{e}\text{k}\text{e}\text{e}\text{p}\text{i}\text{n}\text{g}\:\text{g}\text{e}\text{n}\text{e}\right)$$

$$\:{\Delta\:}{\Delta\:}\text{C}\text{t}\:=\:{\Delta\:}\text{C}\text{t}\:\left(\text{t}\text{r}\text{e}\text{a}\text{t}\text{e}\text{d}\:\text{s}\text{a}\text{m}\text{p}\text{l}\text{e}\right)\--\:{\Delta\:}\text{C}\text{t}\:\left(\text{c}\text{o}\text{n}\text{t}\text{r}\text{o}\text{l}\:\text{a}\text{v}\text{e}\text{r}\text{a}\text{g}\text{e}\right)$$

$$\:{\Delta\:}{\Delta\:}\text{C}\text{t}\:\text{f}\text{o}\text{l}\text{d}\:\text{c}\text{h}\text{a}\text{n}\text{g}\text{e}\:= 2^{\Delta\Delta \text{Ct}} \:\:$$

**Table 2:** The set of primer sequences.

	Forward primer	Reverse primer	Amplicon	Accession no
β-actin	CCGCGAGTACAACCTTCTTG	CAGTTGGTGACAATGCCGTG	297	NM_031144.3
CYP11A1	GCAAAAGGTCTTTGCCTGCG	TGGATTCTGTGTGTGCCGTT	212	NM_017286.3
StAR	TGGCTGCCAAAGACCATCAT	TGGTGGGCAGTCCTTAACAC	241	NM_031558.3
HSD-3β	CTCACATGTCCTACCCAGGC	TATTTTTGAGGGCCGCAAGT	362	NM_001007719.3

### Histopathological examination

Testicular samples from all experimental groups were fixed in 10% neutral buffered formalin (NBF) for 24–48 h. Following fixation, tissues were dehydrated through a graded ethanol series, cleared in xylene, and embedded in paraffin wax using standardized protocols^31^. Serial sections (4–5 μm thickness) were cut using a rotary microtome (Leica RM2235) and mounted on glass slides then stained with hematoxylin and eosin (H&E) for histopathological examination. Stained sections were examined under an Olympus BX43 light microscope equipped with a DP21 digital camera. Representative images were captured at 100×, and 200× magnifications using CellSens Dimension software (Olympus, version 1.16).

Histopathological evaluation of testicular tissues was performed using standardized criteria to ensure objectivity. Spermatogonia, primary spermatocytes, spermatids, and Leydig cells were quantified in 10 randomly selected seminiferous tubule cross-sections per animal (at 400× magnification) by two blind observers. Cell counts were averaged and expressed as cells per tubule. For spermatogenic staging, we adapted the Johnsen score system^32^, which assesses tubular integrity and germ cell presence on a scale of 1–10. Leydig cell populations were evaluated based on cytoplasmic volume and nuclear morphology, as described in^33^.

### Statistical analysis

The data were reported as mean ± standard error (*n* = 5) and *P* < 0.05 was designated as the level of statistical significance. Differences between groups were analyzed using one-way ANOVA followed by Tukey’s post hoc test using the statistical analysis system program (SPSS). A *p*-value < 0.05 was considered statistically significant.

## Results

### Gonadosomatic index (GSI)

**Fig. 2:**
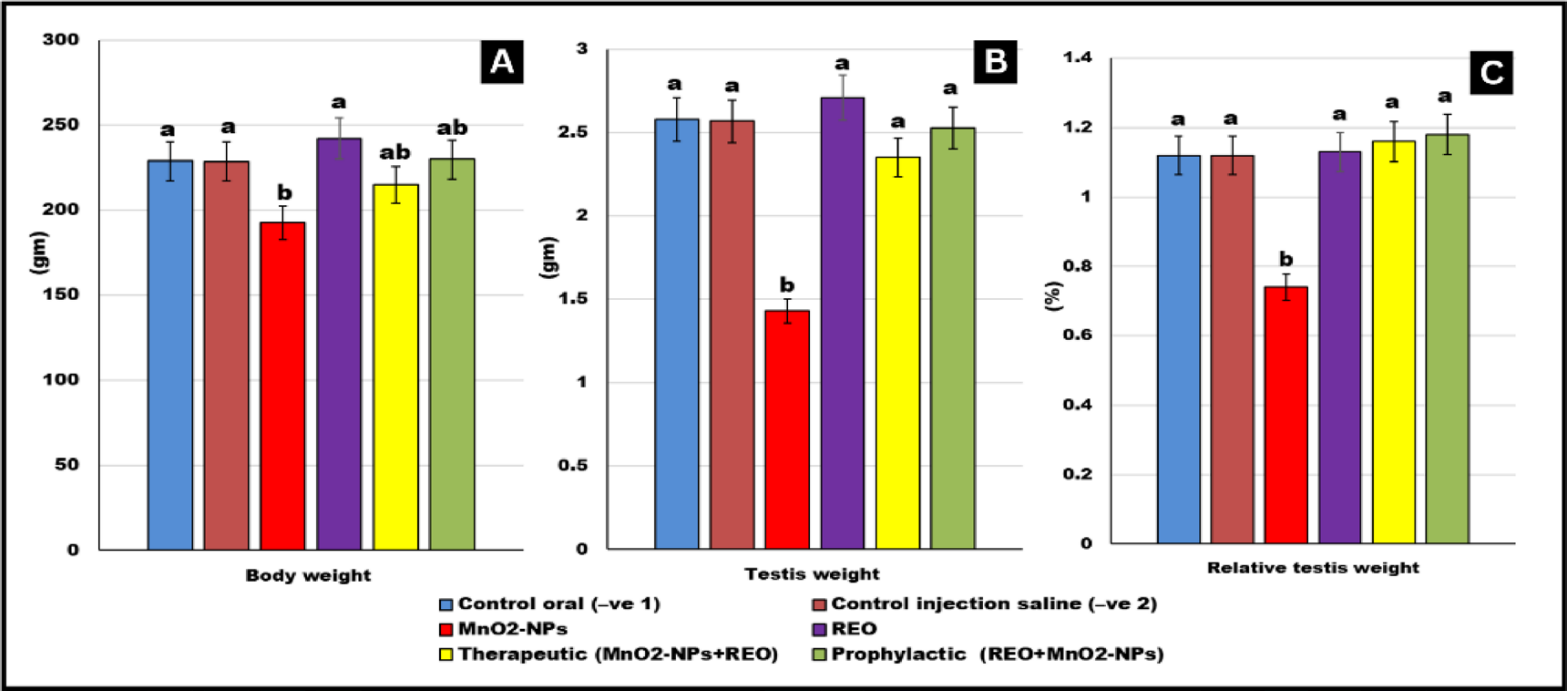
Gonado-somatic index of different experimental groups: Effects of **MnO**_**2**_**-NPs** and **REO** on (**A**) Body weight, (**B**) Testis weight (**C**) Relative Testis weight. Data represented as means ± SE (*n* = 5/replicate). Letters **a**,** b** denote significant differences between groups, ANOVA with Tukey’s post-hoc, *p* < 0.05*). Where control (-ve-1) oral vehicle and control saline (-ve-2) subcutaneous vehicle.

As shown in Fig. 2, dietary exposure to **MnO**_**2**_**-NPs** resulted in a statistically significant reduction in gonadosomatic index (**GSI**) compared to control groups (*p* < 0.05). However, co-administration of rosemary essential oil (**REO**) in both therapeutic **(MnO**_**2**_**-NPs + REO)** and prophylactic **(REO + MnO**_**2**_**-NPs)** regimens effectively attenuated this decline, restoring GSI values to levels comparable with controls. These findings suggest that **REO** supplementation mitigates **MnO**_**2**_**-NPs-**induced gonadal impairment, highlighting its potential protective role in maintaining reproductive organ homeostasis.

### Sperm quality parameters

**Fig. 3:**
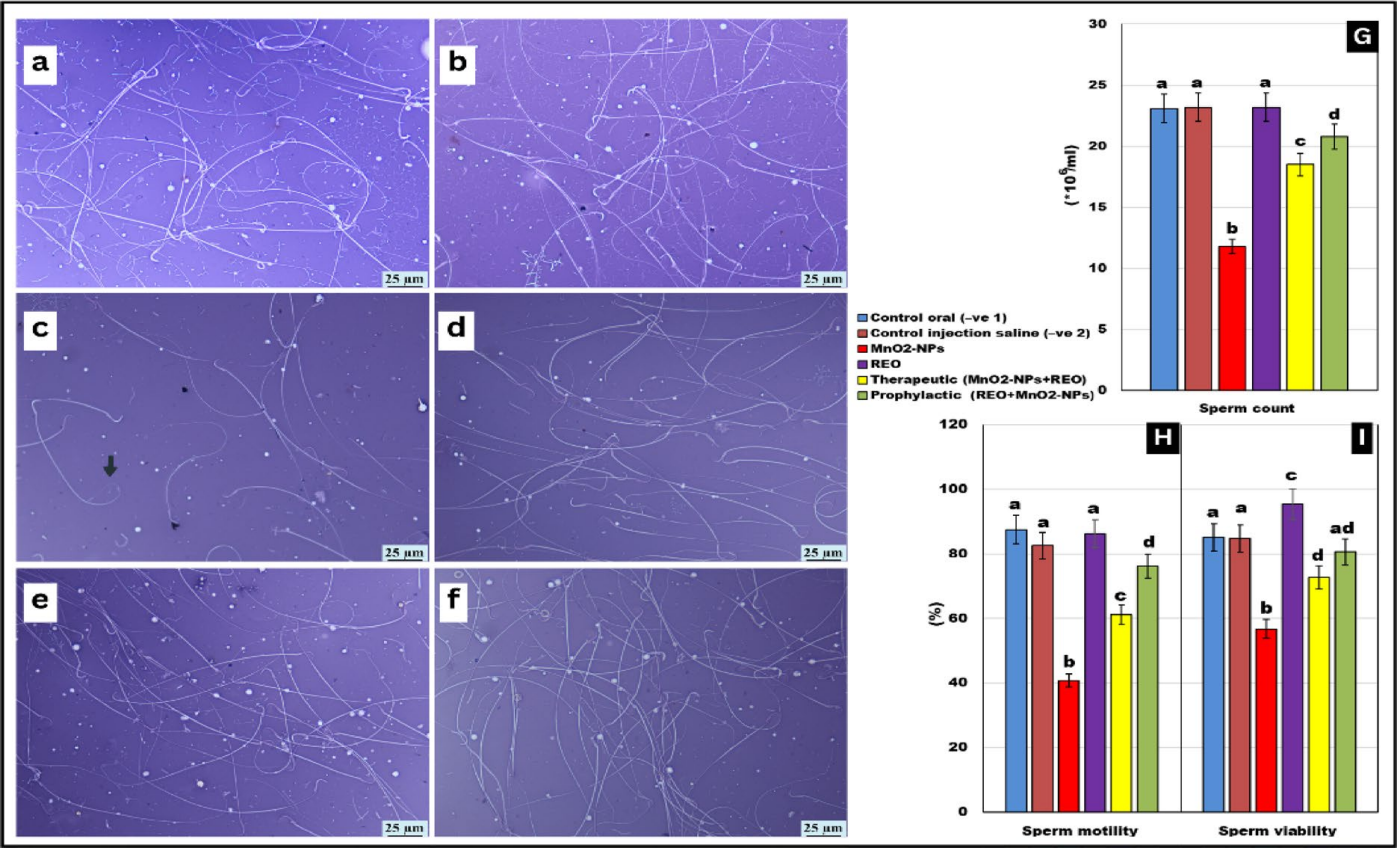
Semen analysis: Effects of **MnO**_**2**_**-NPs** and **REO** on (**a**,**b**) control negative group showing normal sperm. (**c**) **MnO**_**2**_**-NPs** group showing bent tail (black arrow) (**d**) group administered with REO. (**e**) therapeutic group. (**f**) prophylactic group (×40). Smears stained with eosin nigrosine. Graphs (**G**–**I**) represented Sperm count, motility, viability of different experimental groups respectively. Data represented as means ± SE (n = 5/replicate). Letters **a**, **b**, **c**, **d** denote significant differences between groups, ANOVA with Tukey’s post-hoc, p < 0.05*”). Where control -ve 1(oral vehicle) and control saline (-ve 2) subcutaneous vehicle

**Fig. 4:**
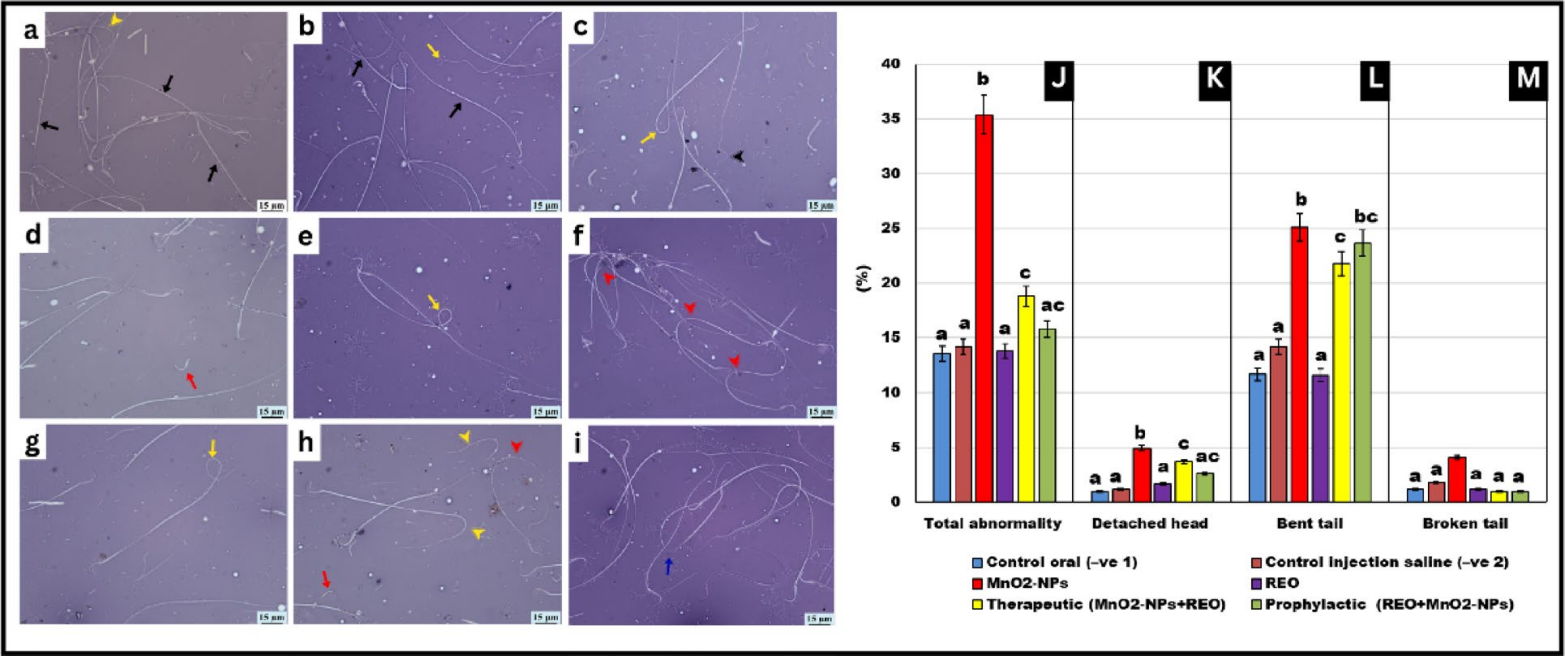
Graphs (**a**–**i**) Sperm abnormalities normal sperm (black arrow), looped tail (yellow arrow), curled tail (black head arrow), detached head (red arrow), bent tail (red arrow-head), curved tail (yellow arrow-head), and broken tail (blue arrow) (15 μm), Graphs (**J**–**M**) Effects of **MnO**_**2**_**-NPs** and **REO** on (**J**) Total abnormalities (%), (**K**) Detached head (%) (**L**) Bent tail (%) (**M**) Broken tail (%) of different experimental groups respectively. Data represented as means ± SE (*n* = 5/replicate). Letters **a**,** b**, **c**, **d** denote significant differences between groups, ANOVA with Tukey’s post-hoc, *p* < 0.05*). Where control (-ve-1) oral vehicle and control saline (-ve-2) subcutaneous vehicle.

As illustrated in Figs. 3 and 4, **MnO**_**2**_**-NP** exposure induced significant adverse effects on sperm quality in treated rats. Compared to untreated controls, the **MnO**_**2**_**-NP** group exhibited marked reductions in sperm motility (↓-56%), viability (↓-36%), and count (↓-31%), alongside a pronounced increase in sperm head and tail morphological abnormalities (*p* < 0.05 for all parameters). Notably, rosemary essential oil **(REO)** co-administration in both prophylactic (**REO** prior to **MnO**_**2**_**-NPs**) and therapeutic (**REO** post **MnO**_**2**_**-NPs**) regimens improved sperm motility, viability, and count, though values in therapeutic groups remained 12% below controls, while the incidence of morphological defects was significantly reduced (*p* < 0.05 vs. **MnO**_**2**_**-NP** group). These results demonstrate that **REO** supplementation preserves sperm integrity and mitigates **MnO**_**2**_**-NPs** effects.

### Serum total antioxidant capacity and testicular antioxidant status

**Fig. 5:**
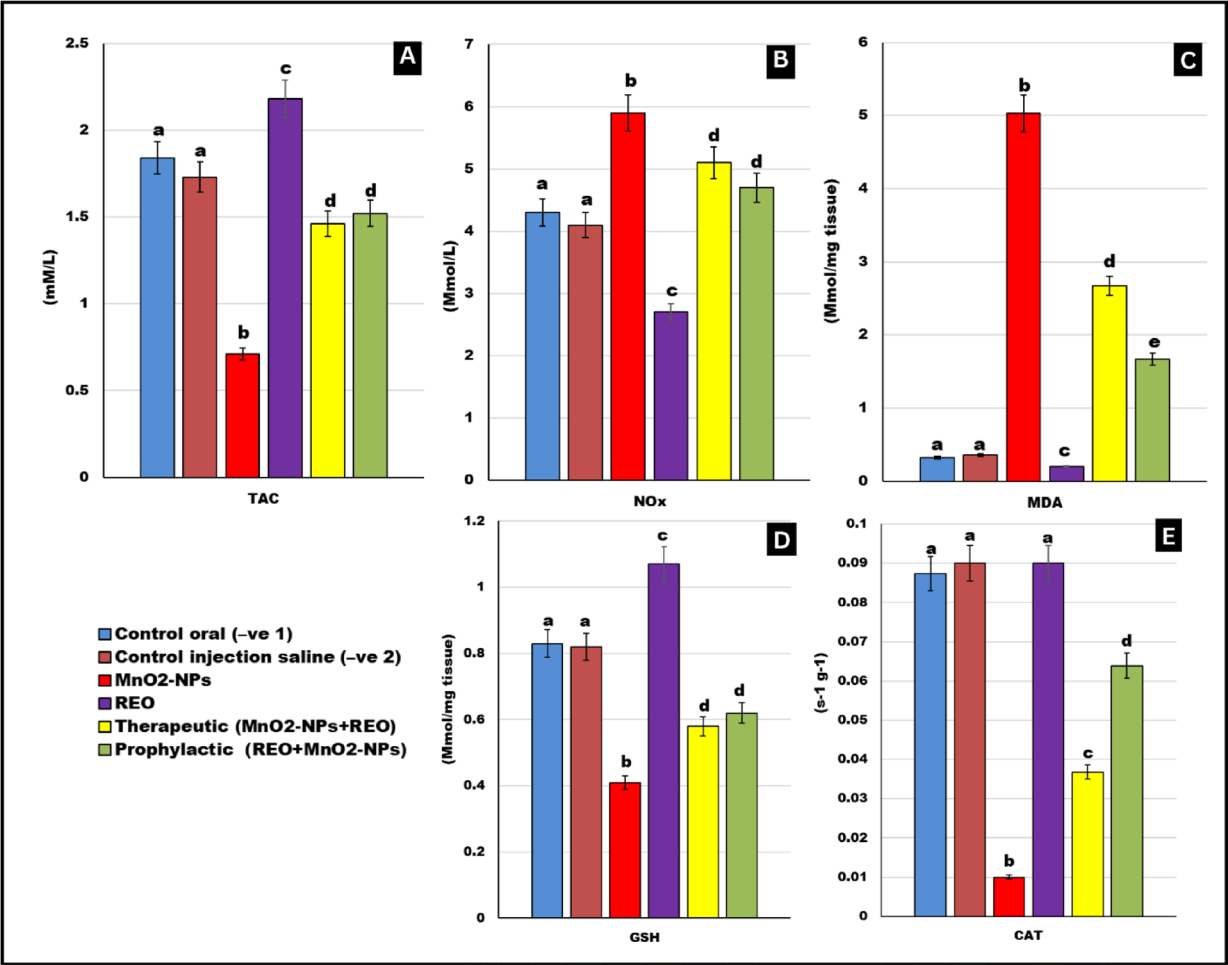
Antioxidant status: Effects of **MnO**_**2**_**-NPs** and **REO** on (**A**) Total antioxidant capacity. (**B**) Nox (**C**) MDA (**E**) GSH (**D**) Catalase levels. Data represented as means ± SE (*n* = 5/replicate). Letters **a**,** b**,** c**,** d**,** e** denote significant differences between groups, ANOVA with Tukey’s post-hoc, *p* < 0.05*). Where control (-ve-1) oral vehicle and control saline (-ve-2) subcutaneous vehicle.

As shown in Fig. 5, **MnO**_**2**_**-NPs** exposure induced pronounced oxidative stress, marked by a significant increase in malondialdehyde (MDA, ↑12.5-fold) and nitric oxide (NO_x_, ↑40%) levels, coupled with a decrease in catalase (CAT, ↓88%), glutathione (GSH, ↓51%), and total antioxidant capacity (TAC, ↓60%) relative to untreated controls (*p* < 0.05). Critically, Co-administration of **REO** (therapeutically or prophylactically) with **MnO**_**2**_**-NPs** attenuated oxidative damage, restoring **MDA** and **NO** concentrations to near-baseline levels while rescuing **CAT**, **GSH**, and **TAC** activity (*p* < 0.05 vs. **MnO**_**2**_**-NP** group). Notably, rosemary essential oil **(REO)** supplementation alone significantly elevated **GSH** (↑130%) and **TAC** (↑125%) levels with significant reduction in **NO**_**x**_ (↓34%), and **MDA** (↓37.5%) compared to controls (*p* < 0.05), suggesting intrinsic antioxidant activity.

### Reproductive hormone levels

**Fig. 6:**
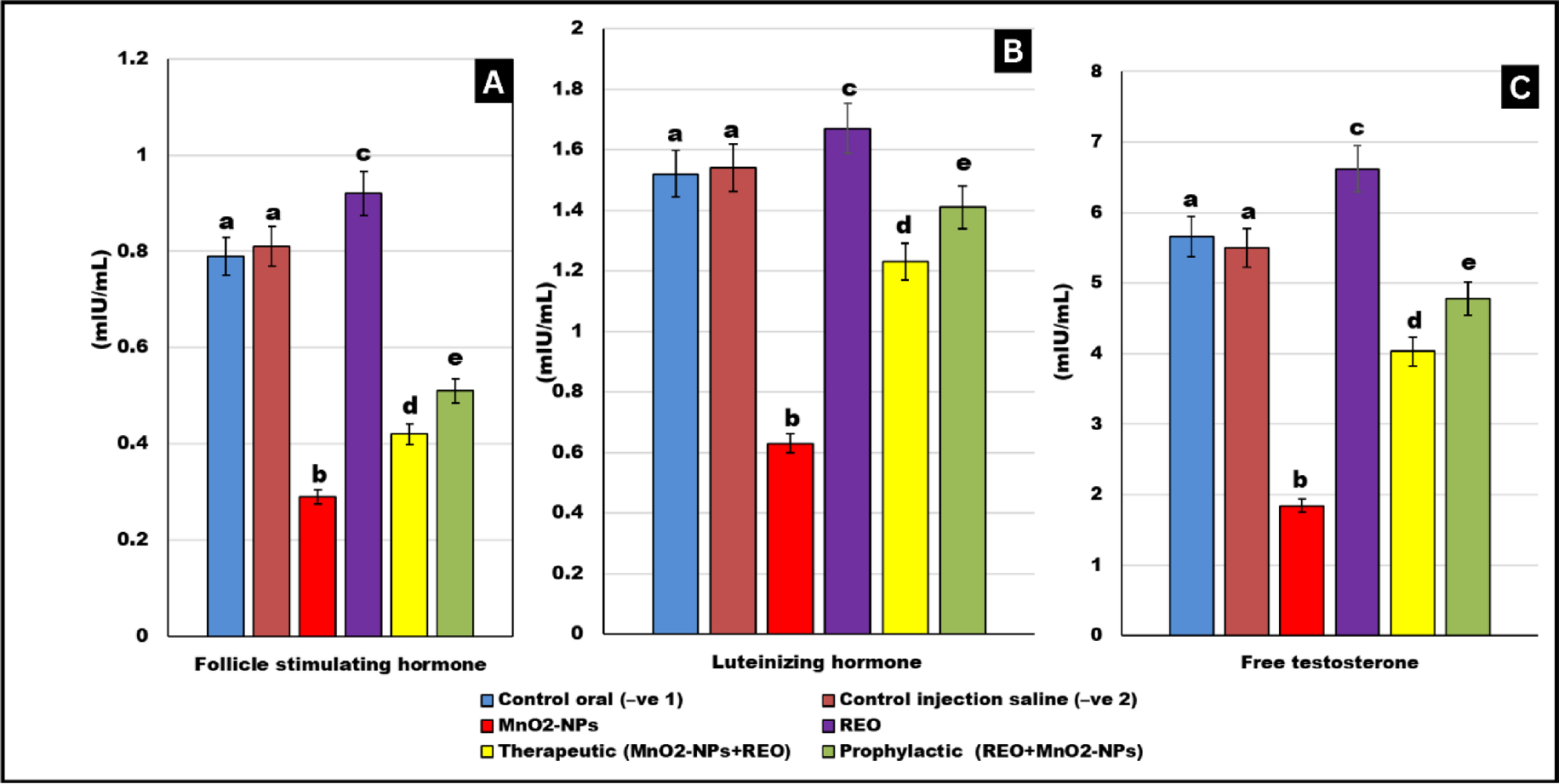
Reproductive hormones: Effects of **MnO**_**2**_**-NPs** and **REO** on (**A**) free testosterone level. (**B**) Follicle-stimulating hormone (FSH), Luteinizing Hormone (LH) Levels. Data represented as means ± SE (*n* = 5/replicate). Letters **a**,** b**,** c**,** d** denote significant differences between groups, ANOVA with Tukey’s post-hoc, *p* < 0.05*). Where control (-ve-1) oral vehicle and control saline (-ve-2) subcutaneous vehicle.

As depicted in Fig. 6, **MnO**_**2**_**-NP**s exposure significantly suppressed serum levels of free testosterone, follicle-stimulating hormone (**FSH**), and luteinizing hormone (**LH**) compared to untreated controls (*p* < 0.05). In contrast, rosemary essential oil (**REO**) supplementation alone significantly elevated free testosterone, **FSH**, and **LH** levels relative to negative controls (*p* < 0.05), underscoring its endocrine-modulatory potential. Both prophylactic (REO pre-treatment) and therapeutic (REO post-MnO2-NP exposure) restoring free testosterone, FSH, and LH concentrations to near-baseline values (*p* < 0.05 vs. **MnO**_**2**_**-NP**s group). These results indicate that **REO** not only enhances endogenous reproductive hormone synthesis under normal conditions but also counteracts **MnO**_**2**_**-NPs**-driven endocrine dysfunction.

### Gene expression analysis

**Fig. 7:**
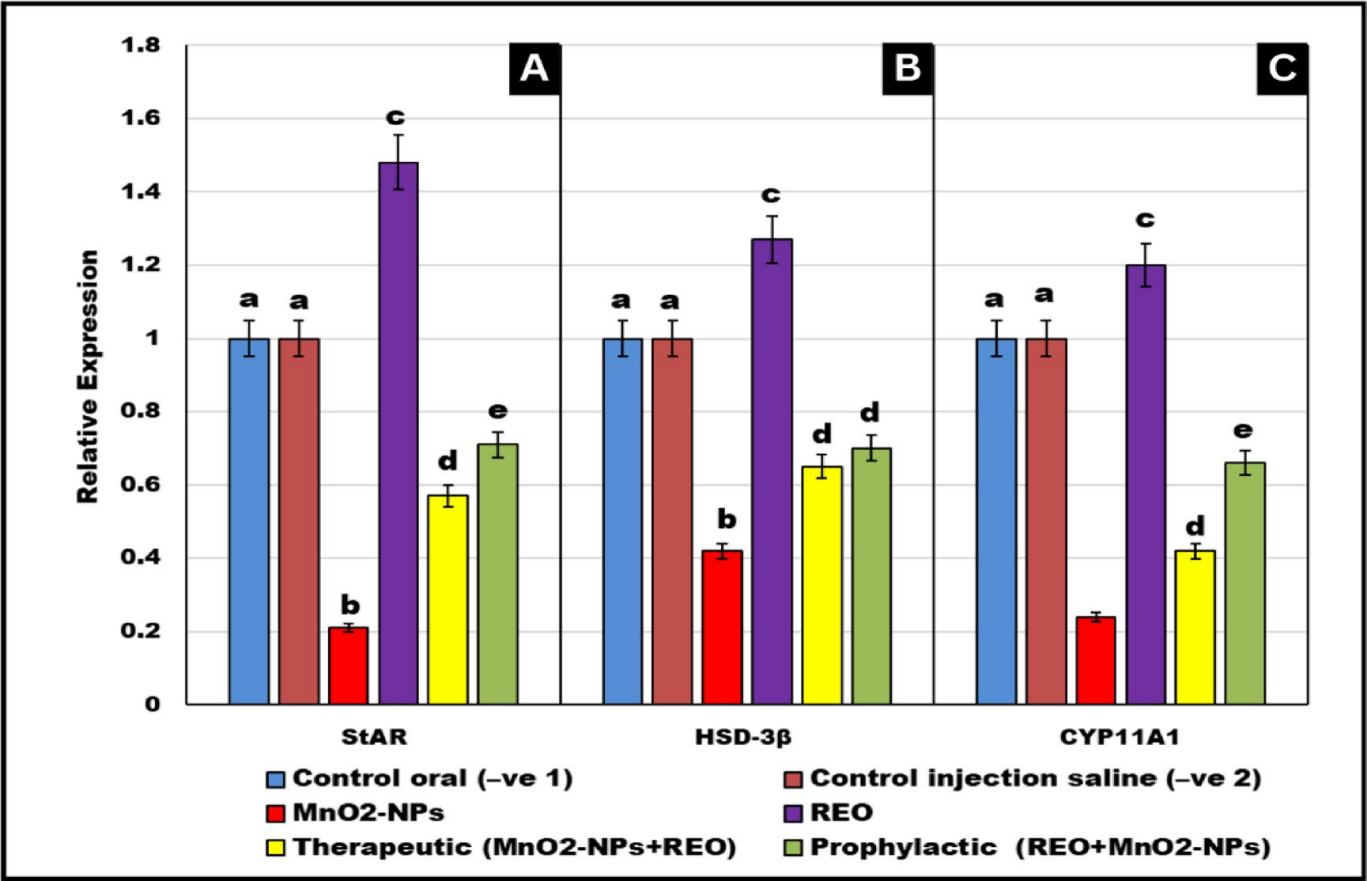
Gene expression analysis: Effects of **MnO**_**2**_**-NPs** and **REO** on (**A**) *StAR*, (**B**) HSD-3β and (**C**) CYP11A1 Levels. Data represented as means ± SE (*n* = 5/replicate). Letters **a**,** b**,** c**,** d**,** e** denote significant differences between groups, ANOVA with Tukey’s post-hoc, *p* < 0.05*). Where control (-ve-1) oral vehicle and control saline (-ve-2) subcutaneous vehicle.

As shown in Fig. 7, **MnO**_**2**_**-NPs** exposure significantly downregulated testicular transcript levels of ***StAR*** (− 79%), **HSD-3β** (− 58%), and **CYP11A1** (− 76%) compared to the negative control groups (*p* < 0.05). In contrast, prophylactic **REO** co-administration restored expression to near-normal levels (85% of control values for ***StAR***, 82% for **HSD-3β**, and 78% for **CYP11A1**), while therapeutic **REO** intervention partially reversed the suppression (62% recovery for ***StAR***, 57% for **HSD-3β**, and 53% for **CYP11A1**). Notably, **REO** alone (without **MnO**_**2**_**-NPs** exposure) upregulated basal expression of ***StAR*** (+ 44%), **HSD-3β** (+ 23%), and **CYP11A1** (+ 20%) relative to controls (*p* < 0.05), suggesting intrinsic stimulatory effects on steroidogenic pathways.

### Histopathological assessment of testicular architecture

**Fig. 8:**
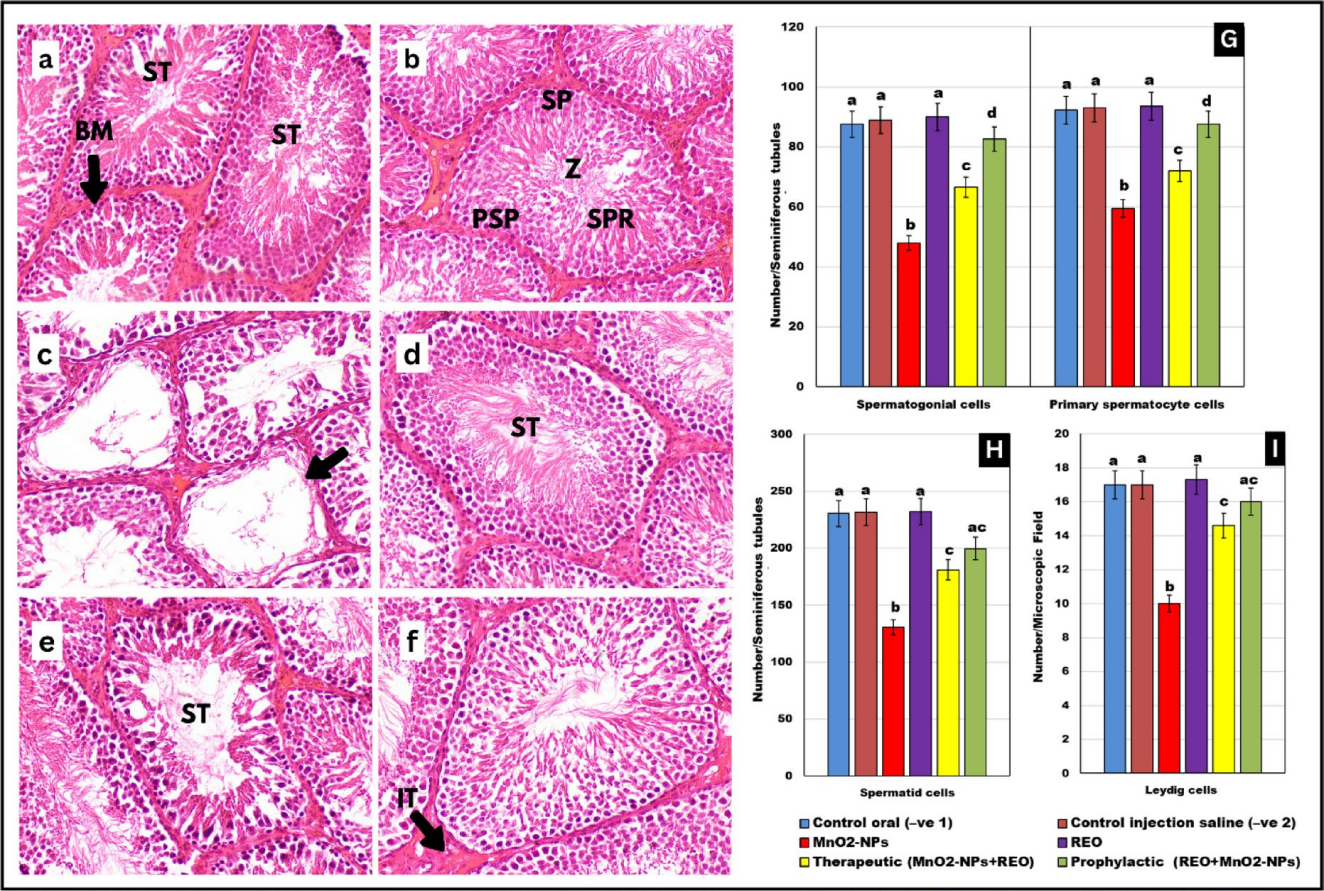
Photomicrograph of testes tissue sections in different groups of rats stained with H&E stain (200x). (**a**,**b**) control groups showing seminiferous tubules **(ST)** exhibit a typical structure, enclosed by a thin and uniform basement membrane **(BM).** The lumina of the tubules are filled with spermatozoa **(Z)**, while the narrow interstitial spaces **(IT)** separate the tubules. The **ST** contain well-organized spermatogenic cords, which consist of spermatogonia **(SP)**, primary spermatocytes **(PSP)**, spermatids **(SPR)**, and Sertoli cells. (**c**) MnO_2_-NPs group showing testicular degeneration with complete absence of germinal cells lining in some seminiferous tubules (arrow) with marked decrease of spermatogenesis, (**d**) rosemary essential oil group showing the normal histological structure with increased diameters of seminiferous tubules, (**e**) therapeutic group, (**f**) protective group showing apparently normal testicular seminiferous tubules with sperm production. Graphs (**G**–**I**) represented Effects of **MnO**_**2**_**-NPs** and **REO** on numbers of spermatogonia cells, primary spermatocyte cell, spermatid cell and Leydig cells. Data represented as means ± SE (*n* = 5/replicate). Letters **a**,** b**,** c**,** d** denote significant differences between groups, ANOVA with Tukey’s post-hoc, *p* < 0.05*). Where control (-ve-1) oral vehicle and control saline (-ve-2) subcutaneous vehicle.

Microscopic evaluation of testicular sections (Fig. 8a–f) revealed distinct histological profiles across experimental groups. In both negative control groups (oral and subcutaneous administration routes), seminiferous tubules exhibited normal architecture, characterized by densely packed spermatogonia, primary spermatocytes, spermatids, Sertoli cells, and abundant luminal spermatozoa, with no evidence of histopathological alterations. Similarly, rosemary essential oil (REO)-supplemented groups demonstrated preserved testicular morphology, comparable to controls, confirming REO’s lack of adverse effects. The therapeutic **REO** group displayed mild but discernible histopathological changes. Some seminiferous tubules exhibited focal vacuolization of the germinal epithelium, accompanied by interstitial vascular congestion (Fig. 8d). However, > 80% of tubules retained near-normal cellular organization, with intact spermatogenic stages. In contrast, the prophylactic **REO** group showed markedly attenuated pathology, with 95% of tubules maintaining structural integrity, a well-defined germinal epithelium, and complete spermatogenic cycles (Fig. 8e).

Quantitative morphometric analysis (Fig. 8G-I) corroborated these observations, **MnO**_**2**_**-NPs**-exposed group exhibited severe germinal epithelial disruption, with significant reductions in spermatogonia (-58%%), primary spermatocytes (-49%), spermatids (-63%), and Leydig cells (-45%) compared to controls (*p* < 0.05). In contrast, therapeutic **REO** administration attenuated these effects, partially restoring spermatogonia (+ 32%), spermatocytes (+ 28%), spermatids (+ 37%), and Leydig cells (+ 24%) though mild vacuolation and vascular congestion persisted. Prophylactic **REO** pre-treatment yielded near-normal histology, with minimal vacuolation, intact germinal epithelium, and robust recovery of spermatogonia (+ 51%), spermatocytes (+ 43%), spermatids (+ 59%), and Leydig cells (+ 40%), closely resembling control levels (*p* < 0.05 vs. **MnO**_**2**_**-NPs** group). These findings collectively demonstrate that **REO** mitigates **MnO**_**2**_**-NPs** induced testicular damage, prophylactic **REO** exposure preserved testicular cytoarchitecture and spermatogenic efficiency more effectively than post-exposure therapeutic intervention.

### Histopathological effects of MnO_2_-NPs on testicular architecture

**Fig. 9:**
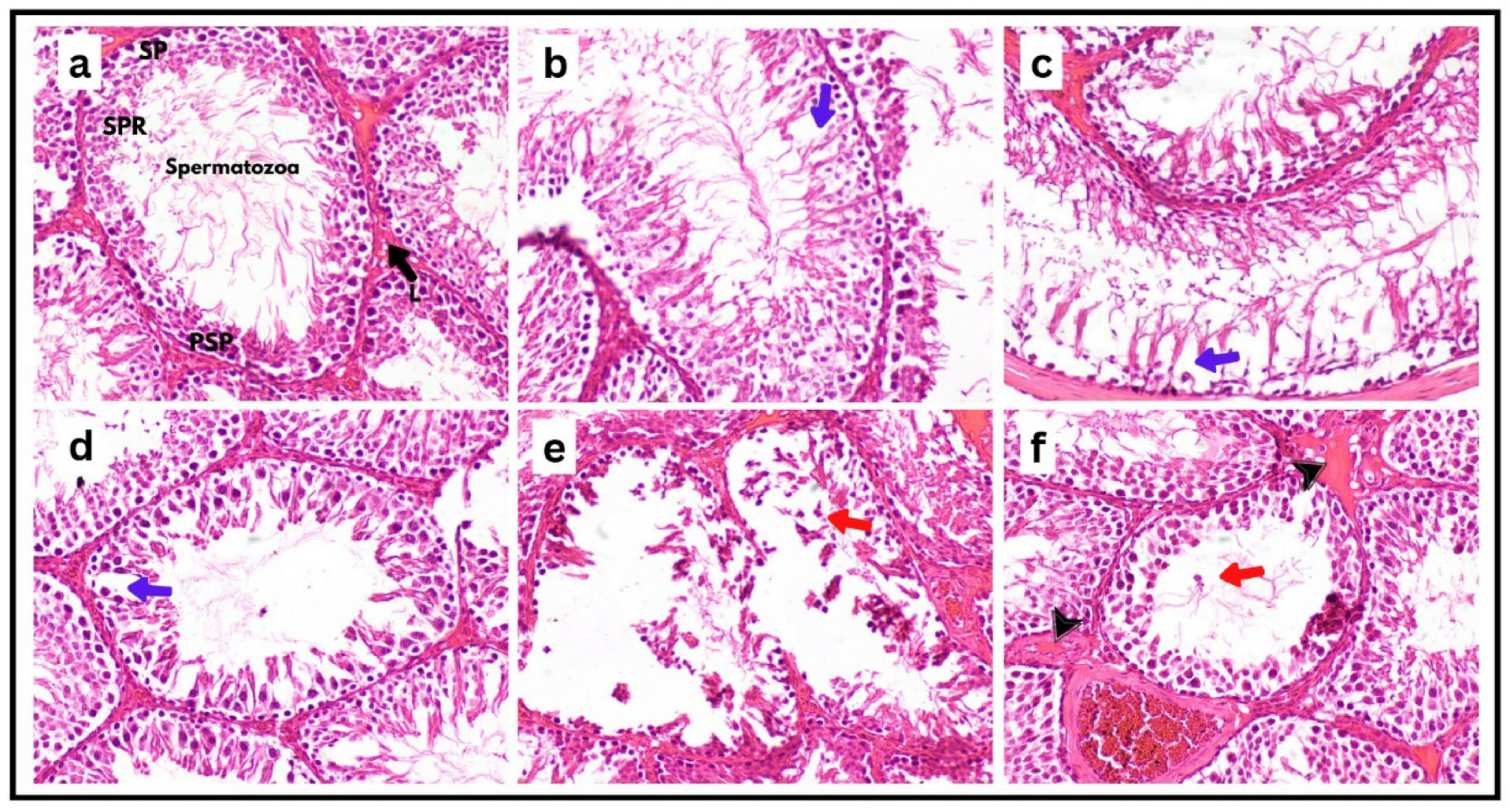
Photomicrograph of testes tissue sections in MnO_**2**_NPs group stained with H&E stain. (200x) showing (**a–c**) vacuolar degeneration with cellular disruption (blue arrow), (**e**,**f**) exfoliation and sloughing of germ cells into the lumen of seminiferous tubules (red arrow), (**f**) interstitial congestion and fluid accumulation (head arrow), where spermatogonia **(SP)**, primary spermatocytes **(PSP)**, spermatids **(SPR)** and Leydig cell **(L)**.

Testicular tissues from **MnO**_**2**_**-NPs**exposed animals exhibited marked histopathological degeneration (Fig. 9). Seminiferous tubules displayed extensive vacuolization, characterized by both microvacuolar and macrovacuolar cytoplasmic lesions within the germinal epithelium (Fig. 9b, c). Germ cell depletion was pronounced, with a reduction in spermatogonia populations compared to controls, and complete absence of spermatids in 30–40% of tubule cross-sections. Disruption of spermatogenic staging was evident, accompanied by frequent exfoliation of immature germ cells into the tubular lumen (Fig. 9d, arrows). Interstitial pathology included severe vascular congestion (increased vascular diameter) and edema, with extracellular fluid accumulation displacing Leydig cell clusters (Fig. 9e). Tubular atrophy quantified by reduced epithelial height. These findings collectively indicate **MnO**_**2**_**-NPs**-induced disruption of spermatogenesis and compromised testicular microarchitecture.

### Morphometric analysis of seminiferous tubules

**Fig. 10:**
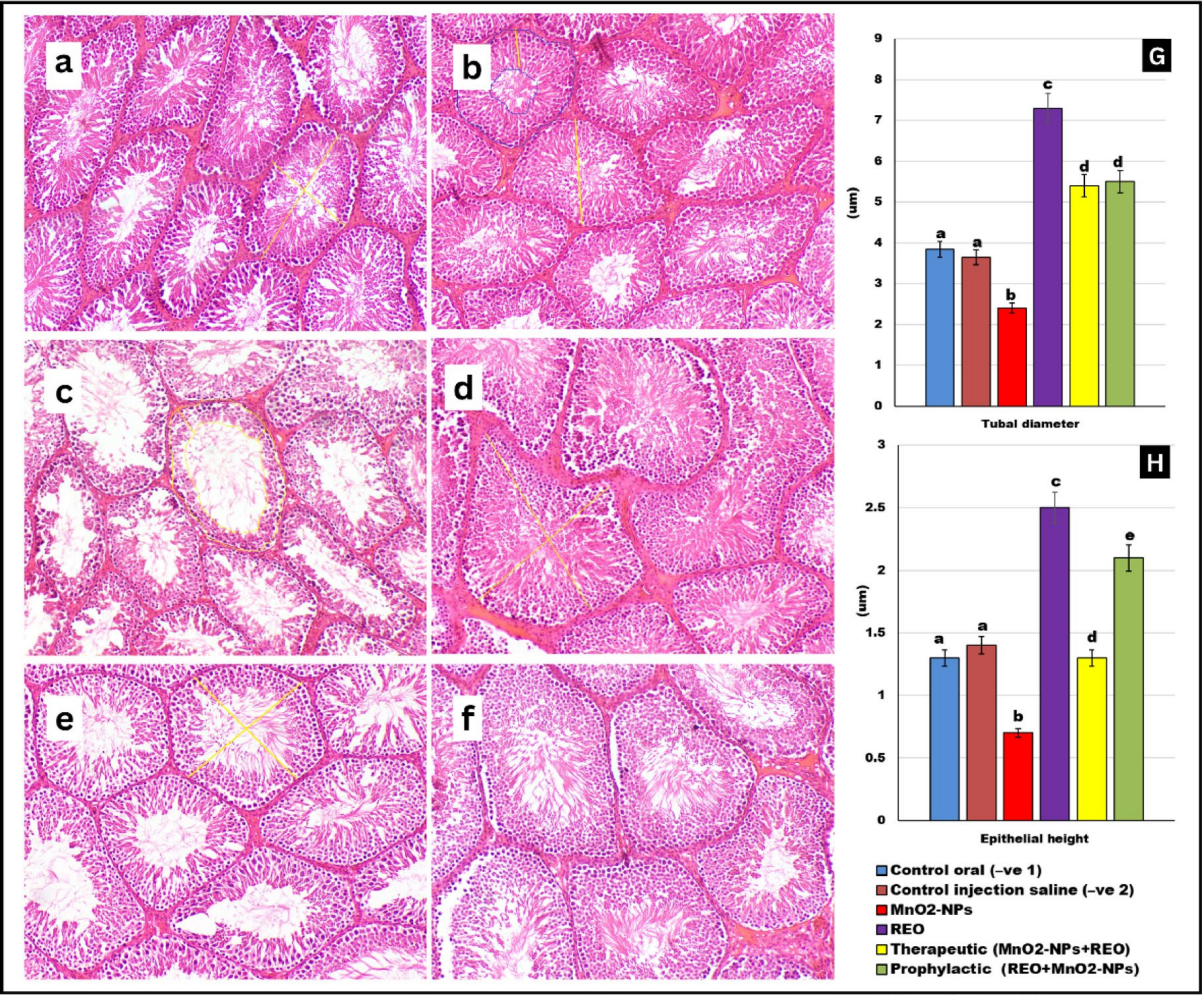
Photomicrograph of testes tissue sections in different groups of rats stained with H&E stain (100x): (**a**,**b**) control groups, (**c**) MnO_2_-NPs group showing decrease in tubular diameter and epithelial height, (**d**) rosemary essential oil group showing increase in tubular diameter and epithelial height, (**e**) therapeutic group, (**fx**( protective group showing an enhancement in tubular diameter and epithelial height, Graphs (**G**,** H**) represent the Effects of **MnO**_**2**_**-NPs** and **REO** on tubal diameter and epithelial height, Data represented as means ± SE (*n* = 5/replicate). Letters **a**,** b**,** c**,** d**,** e** denote significant differences between groups, ANOVA with Tukey’s post-hoc, *p* < 0.05*). Where control (-ve-1) oral vehicle and control saline (-ve-2) subcutaneous vehicle.

Quantitative assessment of testicular morphometry revealed significant alterations in tubular architecture across experimental groups (Fig. 10). Animals exposed to **MnO**_**2**_**-NPs** exhibited a marked reduction in seminiferous tubular diameter and germinal epithelial height, indicative of tubular atrophy and germ cell loss. In contrast, the **REO-**supplemented group showed enhanced diameter tubular parameter and epithelial height exceeding baseline control values, suggesting a pro-spermatogenic effect of REO. Both therapeutic and protective regimens attenuated **MnO**_**2**_**-NPs**-induced morphometric deficits. The prophylactic group demonstrated near-complete restoration of tubular diameter and epithelial height, whereas the therapeutic group showed partial recovery in tubular diameter and epithelial height. These findings align with histopathological observations (Figs. 8 and 9), underscoring **REO’**s capacity to preserve testicular structure, particularly when administered prophylactically.

## Discussion

Manganese plays a dual role in male reproductive health, with essential physiological benefits at optimal levels^34^ but detrimental effects at elevated concentrations. Excessive manganese exposure, particularly via manganese dioxide nanoparticles **MnO**_**2**_**-NPs**, disrupts reproductive function by overwhelming antioxidant defenses, leading to reactive oxygen species **(ROS)** overproduction^35^. Testicular tissue, characterized by high metabolic activity, polyunsaturated fatty acid **(PUFA)** content, and rapid cell division, is exceptionally vulnerable to oxidative damage. **MnO**_**2**_**-NPs**-induced oxidative stress in this study manifested as elevated lipid peroxidation marker, alongside depleted antioxidant enzymes—consistent with prior findings^36^. This redox imbalance correlated with impaired semen quality (reduced sperm count, motility, viability; increased abnormalities) and diminished gonadosomatic index **(GSI)**, reflecting spermatogenic disruption and germ cell loss^37,38^.

Mechanistically, **MnO**_**2**_**-NPs** penetrate the blood-brain barrier, accumulate in the hypothalamus, and disrupt the hypothalamic-pituitary-gonadal **(HPG)** axis, suppressing gonadotropin-releasing hormone **(GnRH)** secretion^39,40^. This GnRH deficiency disrupts pituitary release of luteinizing hormone **(LH)** and follicle-stimulating hormone **(FSH)**, thereby impairing Leydig cell testosterone synthesis—a cascade consistent with the hormonal deficits observed in this study and prior reports^40,41^. Concurrently, **MnO**_**2**_**-NPs** directly inhibit testicular steroidogenesis by downregulating steroidogenic acute regulatory protein ***StAR***, **HSD-3β**,** and CYP11A1**—genes critical for cholesterol transport and testosterone production^8,41,42^. Mitochondrial manganese accumulation exacerbates this by impairing ***StAR-***mediated cholesterol shuttling and disrupting **LH/FSH** signaling. Further molecular disruptions include **NF-κB-**mediated inflammation, which suppresses ***StAR*** via inhibition of **Creb** phosphorylation, and upregulation of **Cox-2/PGE**_**2**_ pathways, impairing **GnRH** neuronal activity^42,43^.

Histopathological analyses revealed **MnO**_**2**_**-NPs**induced testicular degeneration, interstitial hyalinization, and suppressed spermatogenesis, aligning with observed hormonal deficits and germ cell apoptosis^43^. Epididymal dysfunction—marked by weight loss and impaired cell development—underscores systemic reproductive toxicity^37^. Elevated **caspase-3** mRNA levels and reduced vimentin expression in Sertoli cell further indicate disrupted structural support and increased apoptotic signaling^44^. Collectively, excessive manganese exposure disrupts redox balance, hormonal homeostasis, and cellular integrity via oxidative, inflammatory, and apoptotic pathways, culminating in dose-dependent male infertility. These findings emphasize the necessity of regulating manganese exposure to mitigate its reproductive risks.

Rosemary essential oil **(REO)** supplementation effectively mitigated manganese dioxide nanoparticle **MnO**_**2**_**-NPs**induced testicular injury, primarily through the synergistic antioxidant and anti-inflammatory actions of its bioactive constituents, such as **α-pinene**, **β-myrcene**, and **1**,**8-cineole**^45,46^. These monoterpenes attenuated oxidative damage by neutralizing reactive oxygen species **(ROS)** via electron donation, inhibiting lipid peroxidation and enhancing endogenous antioxidant enzyme defenses thereby preserving sperm membrane integrity and mitochondrial function^47,48^. Restoration of the gonadosomatic index **(GSI)** and seminiferous epithelial architecture in **REO-**treated groups underscored rosemary’s capacity to promote germ cell survival and regeneration. This protective effect likely stems from modulation of apoptotic pathways **(caspase-3 suppression)** and stabilization of Sertoli cell vimentin networks, which are critical for structural and functional support during spermatogenesis^48–50^. Furthermore, improvements in sperm motility, count, and viability were attributed to **REO**’s dual role in scavenging **ROS** and shielding spermatogenic cells from oxidative **DNA** damage, thereby maintaining genomic stability and cellular viability^51–53^.

In the present study, **REO** mitigated manganese nanoparticle **(MnO**_**2**_**NP)-**induced reproductive toxicity in mature males by attenuating oxidative stress, restoring steroidogenic function, and preserving hypothalamic-pituitary-gonadal **(HPG)** axis activity. Our results demonstrate that **REO** treatment significantly reduced testicular oxidative damage alongside restored antioxidant enzymatic activity. This antioxidant efficacy is attributable to **REO**’s bioactive constituents, including **rosmarinic acid**,** carnosic acid**,** and α-pinene**, which directly scavenged reactive oxygen species **(ROS)** and inhibited lipid peroxidation. **α-Pinene** attenuated oxidative stress by activating the **Nrf2** pathway, enhancing glutathione synthesis and superoxide dismutase **(SOD)** activity, as demonstrated in cisplatin-induced reproductive toxicity models^54^. **1**,**8-Cineole** suppressed **NF-κB-**mediated inflammation and **COX-2/PGE**_**2**_ production, as shown in cadmium-induced testicular injury^55^, thereby preserving sperm membrane integrity and mitochondrial function^47,51^. Furthermore, **REO** suppressed **caspase-3**-mediated germ cell apoptosis and stabilized vimentin expression in Sertoli cells, correlating with improved seminiferous tubule architecture and spermatogenic recovery^48,56,57^.

Notably, REO restored serum testosterone, **FSH**, and **LH** levels, indicating **HPG** axis stabilization. Mechanistically, this hormonal recovery was driven by enhanced ***StAR*** protein expression and upregulation of key enzymes **(HSD-3β**,** CYP11A1)** in Leydig cells, facilitated by rosemary flavonoids’ activation of the ***cAMP/PKA/Creb*** signalling pathway^58,59^. Concurrently, **REO** attenuated NF-κB-mediated inflammatory signaling, which correlated with reduced systemic inflammation, normalized gonadotropin secretion, and alleviated GnRH neuronal dysfunction, thereby restoring HPG axis activity^60–62^. Histopathological analysis corroborated these findings, showing reduced seminiferous tubule degeneration and interstitial hyalinization in **REO**-treated groups^49,63^.

To our knowledge, this is the first study to demonstrate that the timing of antioxidant intervention critically dictates functional versus structural recovery in nanotoxicity, with prophylactic rosemary essential oil **(REO)** conferring superior protection against **MnO**_**2**_**-NPs**induced testicular damage by bolstering glutathione **(GSH)** reserves, upregulating ***StAR-***mediated steroidogenesis, and mitigating the initial **ROS** surge that disrupts mitochondrial and germ cell integrity. Notably, while therapeutic **REO** partially restored steroidogenic signaling ***(Creb/StAR)*** and attenuated **NF-κB**-driven inflammation but failed to fully reverse structural deficits such as interstitial hyalinization and Sertoli cell vacuolation due to irreversible impact of early oxidative insults on mitotically active germ cells. These insights advocate for integrating prophylactic **REO** into dietary regimens for populations at risk of occupational or environmental **MnO**_**2**_ **NP**s exposure.

### Limitations and future directions

This study has several limitations. First, the exclusive use of male rodents precludes evaluation of sex-specific effects. Second, the dose of REO (250 mg/kg) was selected based on prior studies^14^, but dose-response relationships and pharmacokinetics remain uncharacterized. Third, while oxidative stress markers were assessed, additional endpoints (e.g., DNA fragmentation, apoptotic indices) could strengthen mechanistic claims. Fourth, the prophylactic and therapeutic regimens were tested in a controlled experimental setting, which may not fully replicate chronic occupational exposure scenarios. Future studies should investigate long-term outcomes, individual **REO** constituents, and translational relevance to humans.

## Conclusion

**REO** demonstrates potential in mitigating **MnO**_**2**_**-NPs** induced testicular injury in rodents, primarily via antioxidant activity and partial restoration of steroidogenic pathways. Prophylactic administration showed greater efficacy, suggesting preventive applications in high-risk settings. However, clinical translation requires further investigation into bioavailability, safety, and efficacy in human-relevant models.

## Electronic supplementary material

Below is the link to the electronic supplementary material.

Supplementary Material 1

Supplementary Material 2

Supplementary Material 3

Supplementary Material 4

Supplementary Material 5

## Data Availability

All relevant data, detailed methodologies, and supplementary information necessary to reproduce the study’s results and analyses are fully included within the manuscript.

## Abbreviations

CAT: Catalase
CREs: cAMP response elements
CYP11A1: Cytochrome P450 family 11
DLS: Dynamic light scattering
FSH: Follicle-stimulating hormone
FTIR: Fourier-transform infrared spectroscopy
GC-MS: Gas chromatography–mass spectrometry
GSH: Reduced glutathione
HSD-3β: 3β-hydroxysteroid dehydrogenase
LH: Luteinizing hormone
MDA: Malondialdehyde
MnO_2_-NPs: Manganese oxide nanoparticles
NOx: Nitric oxide
REO: Rosemary essential oil
ROS: Reactive oxygen species
StAR: Steroidogenic acute regulatory protein
TAC: Total antioxidant capacity
TEM: Transmission electron microscopy
XRD: X-ray diffraction

## References

[1] Nel, A., Xia, T., Madler, L. & Li, N. Toxic potential of materials at the nanolevel. *Science*. **311**(5761), 622–627 (2006). 10.1126/science.1114397 16456071

[2] Yousefalizadegan, N., Mousavi, Z., Rastegar, T., Razavi, Y. & Najafizadeh, P. Reproductive toxicity of manganese dioxide in forms of micro-and nanoparticles in male rats. *Int. J. Reprod. Biomed.* **17** (5), 361 (2019). 31435611 10.18502/ijrm.v17i5.4603 PMC6653491

[3] Studer, J. M., Schweer, W. P., Gabler, N. K. & Ross, J. W. Functions of manganese in reproduction. *Anim. Reprod. Sci.* **238**, 106924. 10.1016/j.anireprosci.2022.106924 (2022). 35121412 10.1016/j.anireprosci.2022.106924

[4] Cheng, J., Fu, J. L. & Zhou, Z. C. The inhibitory effects of manganese on steroidogenesis in rat primary Leydig cells by disrupting steroidogenic acute regulatory (StAR) protein expression. *Toxicology*. **187** (2–3), 139–148 (2003). 12699903 10.1016/s0300-483x(03)00063-5

[5] Cheng, T. M. et al. Toxicologic concerns with current medical nanoparticles. *Int. J. Mol. Sci.* **23**(14), 7597 (2022). 10.3390/ijms23147597 PMC9322368 35886945

[6] Qi, Z. et al. Protective role of m6A binding protein YTHDC2 on CCNB2 in manganese-induced spermatogenesis dysfunction. *Chemico-Biol. Interact.* **351**, 109754. 10.1016/j.cbi.2021.109754 (2022). 10.1016/j.cbi.2021.109754 34822792

[7] Miller, W. L. Steroidogenesis: unanswered questions. *Trends Endocrinol. Metab*. **28** (11), 771–793 (2017). 29031608 10.1016/j.tem.2017.09.002

[8] Negahdary, M., Arefian, Z., Dastjerdi, H. A. & Ajdary, M. Toxic effects of Mn2O3 nanoparticles on rat testis and sex hormone. *J. Nat. Sci. Biol. Med.* **6**(2), 335 (2015). 10.4103/0976-9668.159998 PMC4518404 26283824

[9] Ghasemzadeh Rahbardar, M. & Hosseinzadeh, H. Toxicity and safety of Rosemary (*Rosmarinus officinalis*): a comprehensive review. *Naunyn-Schmiedeberg’s Arch. Pharmacol.* 1–15 (2024). 10.1007/s00210-024-03336-9 39096378

[10] Saied, M., Ali, K. & Mosayeb, A. Rosemary (*Rosmarinus officinalis* L.) essential oil alleviates testis failure induced by Etoposide in male rats. *Tissue Cell.* **81**, 102016 (2023). 36640564 10.1016/j.tice.2023.102016

[11] Infantino, V. et al. Brain mitochondria as a therapeutic target for carnosic acid. *J. Integr. Neurosci.* **23**(3), 53 (2024). 10.31083/j.jin2303053 38538219

[12] Ali, M. E., Zainhom, M. Y., Monir, A., Awad, A. A. E. & Al-Saeed, F. A. Dietary supplementation with Rosemary essential oil improves genital characteristics, semen parameters and testosterone concentration in Barki Rams. *J. Basic. Appl. Zool.* **85** (1), 1–11 (2024).

[13] Nusier, M. K., Bataineh, H. N. & Daradkah, H. M. Adverse effects of Rosemary (*Rosmarinus officinalis* L.) on reproductive function in adult male rats. *Exp. Biol. Med.* **232** (6), 809–813 (2007). 17526773

[14] National Research Council (NRC). *Nutrient Requirements of Laboratory Animals*, 4th edn (National Academies, 1995).

[15] Waller, S. B. et al. Can the essential oil of rosemary (*Rosmarinus officinalis* Linn.) protect rats infected with itraconazole-resistant Sporothrix brasiliensis from fungal spread? *J. Med. Mycol.* **31**(4), 101199 (2021). 10.1016/j.mycmed.2021.101199 34418685

[16] Manjula, R., Thenmozhi, M., Thilagavathi, S., Srinivasan, R. & Kathirvel, A. J. M. T. P. Green synthesis and characterization of manganese oxide nanoparticles from Gardenia resinifera leaves. *Mater. Today Proc.* **26**, 3559–3563 (2020).

[17] Nagate, T. et al. Diluted isoflurane as a suitable alternative for diethyl ether for rat anaesthesia in regular toxicology studies. *J. Vet. Med. Sci.* **69** (11), 1137–1143 (2007). 18057828 10.1292/jvms.69.1137

[18] Adebayo, A. O., Oke, B. O. & Akinloye, A. K. Characterizing the gonadosomatic index and its relationship with age in greater cane rat (Thryonomys swinderianus, Temminck). *J. Vet. Anat.* **2** (2), 53–59 (2009).

[19] Seed, J. et al. Methods for assessing sperm motility, morphology, and counts in the rat, rabbit, and dog: a consensus report. *Reprod. Toxicol.* **10** (3), 237–244. 10.1016/0890-6238(96)00028-7 (1996). 8738562 10.1016/0890-6238(96)00028-7

[20] Ohkawa, H., Ohishi, N. & Yagi, K. Assay for lipid peroxides in animal tissues by thiobarbituric acid reaction. *Anal. Biochem.* **95** (2), 351–358. 10.1016/0003-2697(79)90738-3 (1979). 36810 10.1016/0003-2697(79)90738-3

[21] Koracevic, D., Koracevic, G., Djordjevic, V., Andrejevic, S. & Cosic, V. Method for the measurement of antioxidant activity in human fluids. *J. Clin. Pathol.* **54** (5), 356–361 (2001). 11328833 10.1136/jcp.54.5.356 PMC1731414

[22] Aebi, H. Catalase in vitro. In *Methods in Enzymology*, vol. 105, 121–126 (Academic, 1984). 10.1016/s0076-6879(84)05016-3 6727660

[23] Ellman, G. L. Tissue sulfhydryl groups. *Arch. Biochem. Biophys.* **82** (1), 70–77 (1959). 13650640 10.1016/0003-9861(59)90090-6

[24] Albro, P. W., Corbett, J. T. & Schroeder, J. L. Application of the thiobarbiturate assay to the measurement of lipid peroxidation products in microsomes. *J. Biochem. Biophys. Methods*. **13** (3), 185–194 (1986). 3782721 10.1016/0165-022x(86)90092-8

[25] Miranda, K. M., Espey, M. G. & Wink, D. A. A rapid, simple spectrophotometric method for simultaneous detection of nitrate and nitrite. *Nitric Oxide*. **5** (1), 62–71 (2001). 11178938 10.1006/niox.2000.0319

[26] Tietz, N. W. Clinical guide to laboratory tests. In *Clinical Guide to lLaboratory Tests*, 1096–1096 (1995).

[27] Uotila, M., Ruoslahti, E. & Engvall, E. Two-site sandwich enzyme immunoassay with monoclonal antibodies to human alpha-fetoprotein. *J. Immunol. Methods*. **42** (1), 11–15 (1981). 6165775 10.1016/0022-1759(81)90219-2

[28] Rozen, S. & Skaletsky, H. Primer3 on the WWW for general users and for biologist programmers. *Bioinform. Methods Protoc.* 365–386 (1999). 10.1385/1-59259-192-2:365 10547847

[29] Andersen, C. L., Jensen, J. L. & Ørntoft, T. F. Normalization of real-time quantitative reverse transcription-PCR data: a model-based variance Estimation approach to identify genes suited for normalization, applied to bladder and colon cancer data sets. *Cancer Res.* **64** (15), 5245–5250 (2004). 15289330 10.1158/0008-5472.CAN-04-0496

[30] Livak, K. J. & Schmittgen, T. D. Analysis of relative gene expression data using real-time quantitative PCR and the 2 – ∆∆CT method. *Methods*. **25**(4), 402–408. 10.1006/meth.2001.1262 (2001). 10.1006/meth.2001.1262 11846609

[31] Bancroft, J. D. & Gamble, M. *Theory and Practice of Histological Techniques* (Elsevier Health Sciences, 2008).

[32] Johnsen, S. G. Testicular biopsy score count–a method for registration of spermatogenesis in human testes: normal values and results in 335 hypogonadal males. *Hormone Res. Paediatr*. **1** (1), 2–25 (1970). 10.1159/000178170 5527187

[33] Haider, S. G. Cell biology of Leydig cells in the testis. *Int. Rev. Cytol.* **233** (4), 181–241 (2004). 15037365 10.1016/S0074-7696(04)33005-6

[34] Studer, J. M., Schweer, W. P., Gabler, N. K. & Ross, J. W. Functions of manganese in reproduction. *Anim. Reprod. Sci.* **238**, 106924 (2022). 10.1016/j.anireprosci.2022.106924 35121412

[35] Souza, T. L. et al. Evaluation of Mn exposure in the male reproductive system and its relationship with reproductive dysfunction in mice. *Toxicology*. **441**, 152504 (2020). 32445656 10.1016/j.tox.2020.152504

[36] Wang, Y., Fu, X. & Li, H. Mechanisms of oxidative stress-induced sperm dysfunction. *Front. Endocrinol.* **16**, 1520835 (2025). 10.3389/fendo.2025.1520835 PMC11835670 39974821

[37] Takeshima, T. et al. Oxidative stress and male infertility. *Reprod. Med. Biol*. **20** (1), 41–52 (2021). 33488282 10.1002/rmb2.12353 PMC7812476

[38] Pardhiya, S. et al. Cumulative effects of manganese nanoparticle and radiofrequency radiation in male Wistar rats. *Drug Chem. Toxicol.* **45** (3), 1395–1407 (2022). 33111595 10.1080/01480545.2020.1833905

[39] Boudou, F., Aldi, D. E. H., Slimani, M. & Berroukche, A. The impact of chronic exposure to manganese on testiculaire tissue and sperm parameters in rat Wistar. *Int. J. Nat. Sci. Res.* **3**, 12–19 (2014).

[40] Owumi, S. E., Danso, O. F. & Nwozo, S. O. Gallic acid and omega-3 fatty acids mitigate epididymal and testicular toxicity in manganese‐treated rats. *Andrologia*. **52** (7), e13630 (2020). 32396264 10.1111/and.13630

[41] Adedara, I. A., Abolaji, A. O., Awogbindin, I. O. & Farombi, E. O. Suppression of the brain-pituitary-testicular axis function following acute arsenic and manganese co-exposure and withdrawal in rats. *J. Trace Elem. Med Biol.* **39**, 21–29 (2017). 27908416 10.1016/j.jtemb.2016.07.001

[42] Manna, P. R., Dyson, M. T. & Stocco, D. M. Regulation of the steroidogenic acute regulatory protein gene expression: present and future perspectives. *Mol. Hum. Reprod.* **15** (6), 321–333 (2009). 19321517 10.1093/molehr/gap025 PMC2676994

[43] Stocco, D. M. & Sodeman, T. C. The 30-kDa mitochondrial proteins induced by hormone stimulation in MA-10 mouse Leydig tumor cells are processed from larger precursors. *J. Biol. Chem.* **266** (29), 19731–19738 (1991). 1918079

[44] Cai, X. L., Wang, G. & Guo, H. Effect of manganese on caspase-3 mRNA regulation in spermatogenic cell and the expression of vimentin on Sertoli cell in rats. *Acta Anat. Sin*. **41**, 400–404 (2010).

[45] Al-Rikaby, A. A. Evaluating the influence of Rosemary leaves extract on hormonal and histopathological alterations in male rabbits exposed to Cypermethrin. *Arch. Razi Inst.* **78** (3), 797 (2023). 38028826 10.22092/ARI.2022.359859.2487 PMC10657936

[46] Rašković, A. et al. Antioxidant activity of Rosemary (*Rosmarinus officinalis* L.) essential oil and its hepatoprotective potential. *BMC Complement. Altern. Med.* **14**, 1–9 (2014). 25002023 10.1186/1472-6882-14-225 PMC4227022

[47] Liu, S. et al. Carnosic acid prevents heat stress-induced oxidative damage by regulating heat-shock proteins and apoptotic proteins in mouse testis. *Biol. Chem.* **405** (11–12), 745–749 (2024). 39630978 10.1515/hsz-2023-0374

[48] Tousson, E., Bayomy, M. F. & Ahmed, A. A. Rosemary extract modulates fertility potential, DNA fragmentation, injury, KI67 and P53 alterations induced by Etoposide in rat testes. *Biomed. Pharmacother.* **98**, 769–774 (2018). 29571245 10.1016/j.biopha.2018.01.025

[49] Da Conceicao Fernandes, I. et al. Exploring the antioxidant properties of rosmarinus officinalis essential oil and its traditional applications: a scope analysis. *Revista Científica Da Faminas*. **19** (1), 112–128 (2024).

[50] Hajhosseini, L., Khaki, A., Merat, E. & Ainehchi, N. Effect of Rosmarinic acid on Sertoli cells apoptosis and serum antioxidant levels in rats after exposure to electromagnetic fields. *Afr. J. Tradit. Complement. Altern. Med.* **10** (6), 477–480 (2013). 24311872 10.4314/ajtcam.v10i6.14 PMC3847387

[51] Abdel-Daim, M. M., Mohamed, H. A. & Alkhamees, H. A. Impact of *Rosmarinus officinalis* L. on male reproductive function and sperm quality in rats. *Environ. Toxicol. Pharmacol.* **54**, 123–129. 10.1016/j.etap.2017.07.002 (2017).

[52] Aghamiri, S. M., Eslami Farsani, M., Seyedebrahimi, R., Sarikhani, M. J. & Ababzadeh, S. Synergic effects of Rosemary extract and aerobic exercise on sperm parameters and testicular tissue in an aged rat model. *Gene Cell. Tissue* (2022).

[53] Turk, G. et al. Dietary Rosemary oil alleviates heat stress-induced structural and functional damage through lipid peroxidation in the testes of growing Japanese quail. *Anim. Reprod. Sci.* **164**, 133–143 (2016). 26656503 10.1016/j.anireprosci.2015.11.021

[54] Demir, S. et al. Alpha-pinene neutralizes cisplatin-induced reproductive toxicity in male rats through activation of Nrf2 pathway. *Int. Urol. Nephrol.* **56** (2), 527–537 (2024). 37789204 10.1007/s11255-023-03817-5

[55] De Oliveira, M. G. et al. 1,8-Cineole attenuates oxidative stress and testicular damage in rats exposed to cadmium: role of NF-κB/MAPK Inhibition and Nrf2 activation. *Biomed. Pharmacother.* **105**, 555–565 (2018).

[56] Oze, O., Akinmoladun, F. O., Ademiluyi, A. O. & Akinmoladun, A. F. Effect of Rosemary extract on sperm morphology and quality in male rats. *J. Ethnopharmacol.* **211**, 233–240. 10.1016/j.jep.2017.09.030 (2018).

[57] Hu, X. et al. The consequence and mechanism of dietary flavonoids on androgen profiles and disorders amelioration. *Crit. Rev. Food Sci. Nutr.* **63** (32), 11327–11350 (2023). 35796699 10.1080/10408398.2022.2090893

[58] Li, W. et al. Effects of apigenin on steroidogenesis and steroidogenic acute regulatory gene expression in mouse Leydig cells. *J. Nutr. Biochem.* **22** (3), 212–218 (2011). 20537519 10.1016/j.jnutbio.2010.01.004 PMC2939222

[59] King, S. R. & LaVoie, H. A. Gonadal transactivation of STARD1, CYP11A1 and HSD3B. *Front. Biosci.* **17** (1), 824–846 (2012). 10.2741/3959 22201776

[60] Habtemariam, S. Anti-inflammatory therapeutic mechanisms of natural products: insight from Rosemary diterpenes, carnosic acid and carnosol. *Biomedicines*. **11** (2), 545 (2023). 36831081 10.3390/biomedicines11020545 PMC9953345

[61] Alagawany, M. et al. Rosmarinic acid: modes of action, medicinal values and health benefits. *Anim. Health Res. Rev.* **18** (2), 167–176 (2017). 29110743 10.1017/S1466252317000081

[62] Chen, W. P. et al. Rosmarinic acid down-regulates NO and PGE 2 expression via MAPK pathway in rat chondrocytes. *J. Cell. Mol. Med.* **22** (1), 346–353 (2018). 28945000 10.1111/jcmm.13322 PMC5742733

[63] Cormier, M. et al. Influences of flavones on cell viability and cAMP-dependent steroidogenic gene regulation in MA-10 Leydig cells. *Cell Biol. Toxicol.* **34**, 23–38 (2018). 28455626 10.1007/s10565-017-9395-8

