# Supplementary figures and images for: Rosemary essenitial oil counters MnO2 nanoparticle-induced fertility deficits in rats via antioxidant mechanisms and upregulation of StAR signalling
Source: Sci Rep. 2025 Jun 20;15:20201. doi: 10.1038/s41598-025-06345-7 (PMC12181236; doi:10.1038/s41598-025-06345-7)

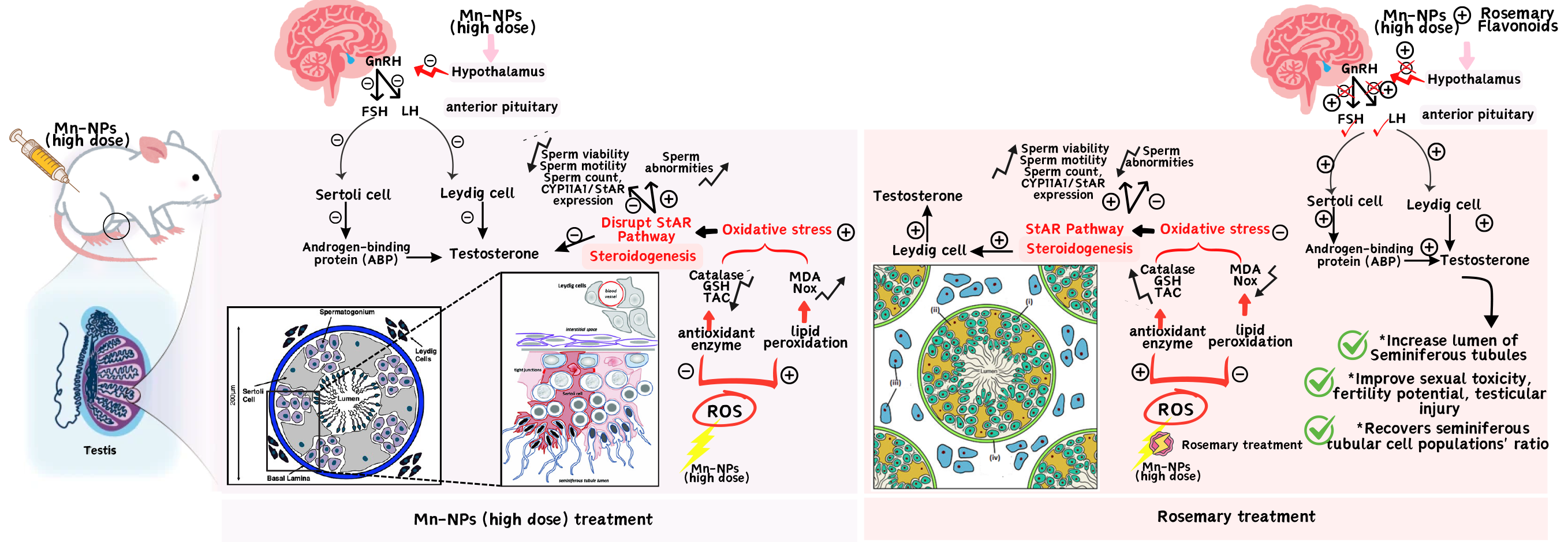

Supplement: Supplementary file 1 — Supplementary Material 1 [file 41598_2025_6345_MOESM1_ESM.png]

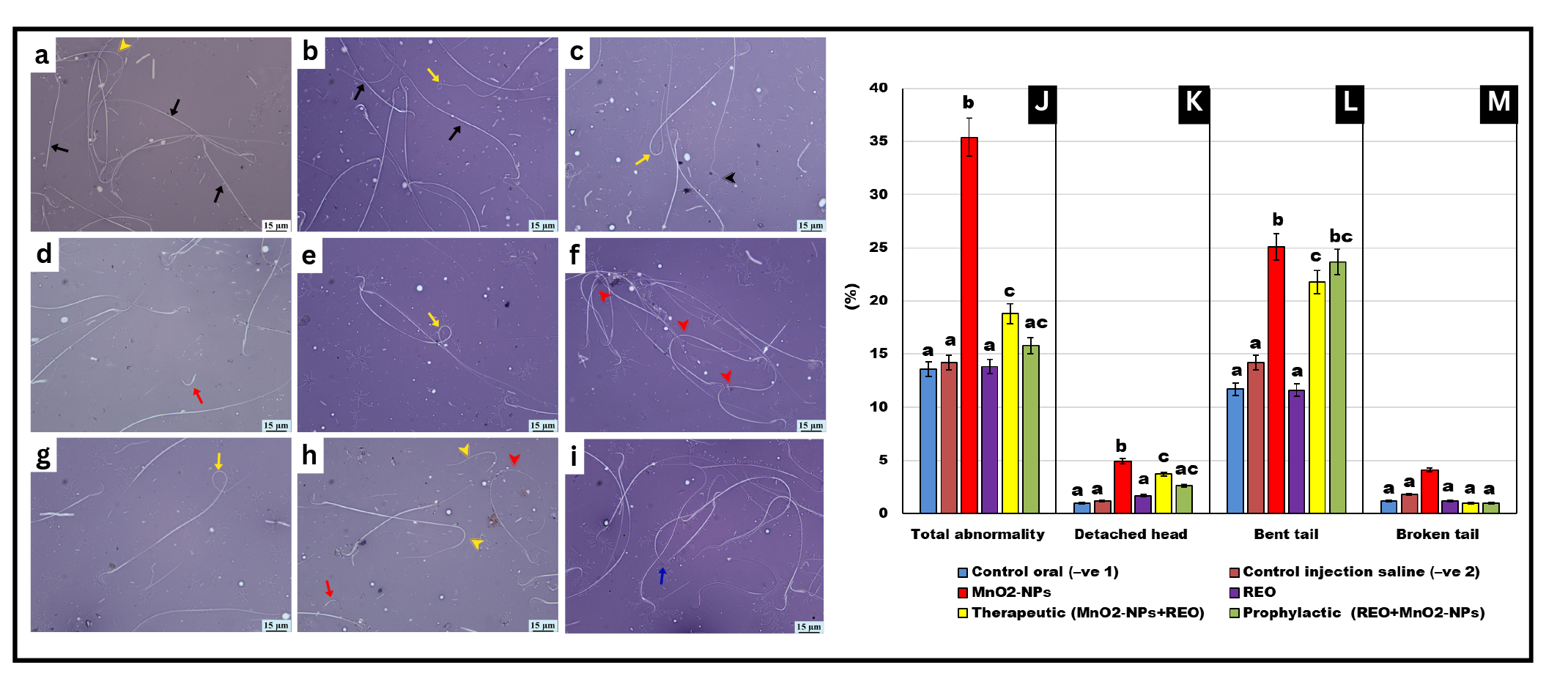

Supplement: Supplementary file 3 — Supplementary Material 3 [file 41598_2025_6345_MOESM3_ESM.png]

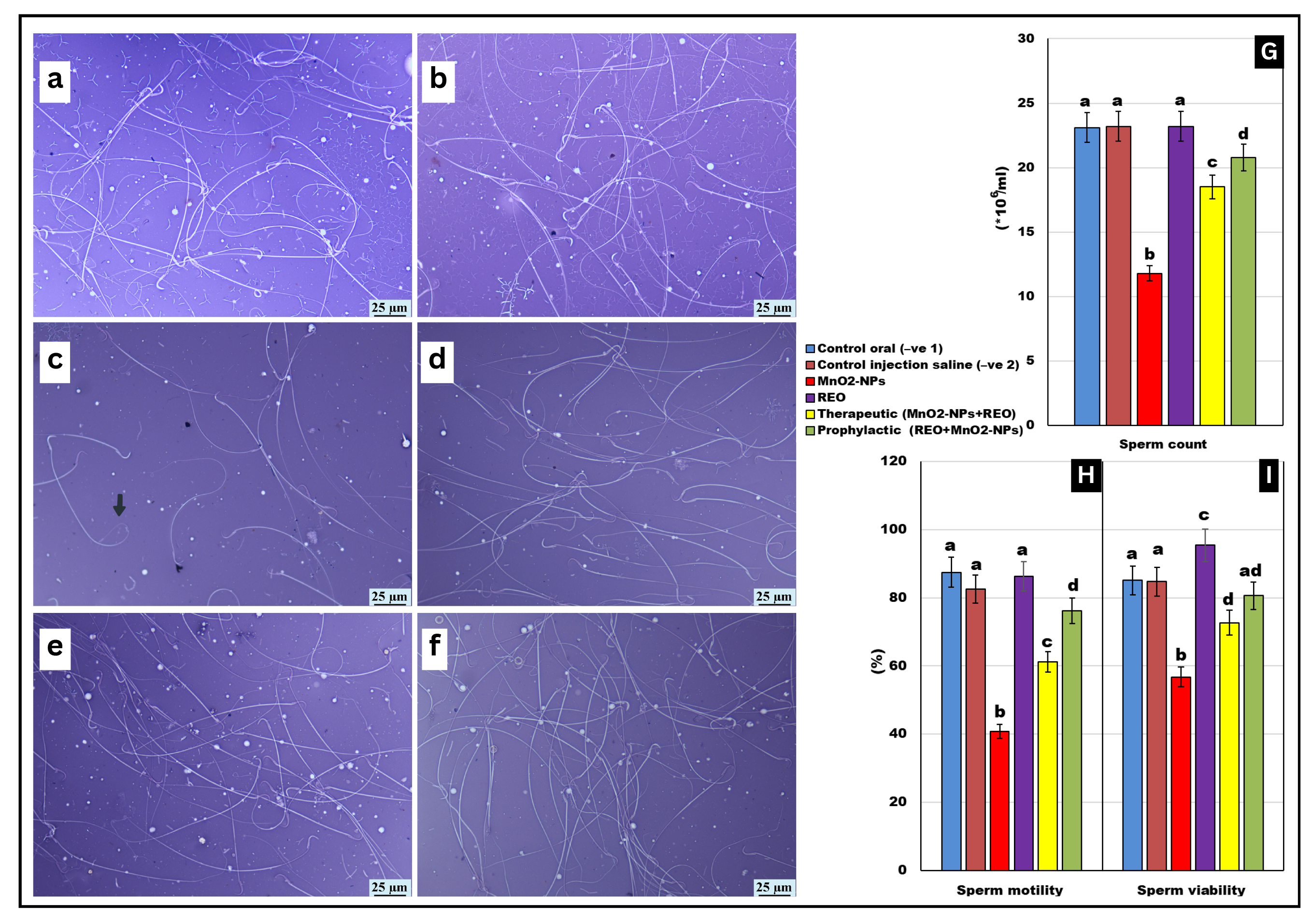

Supplement: Supplementary file 4 — Supplementary Material 4 [file 41598_2025_6345_MOESM4_ESM.png]

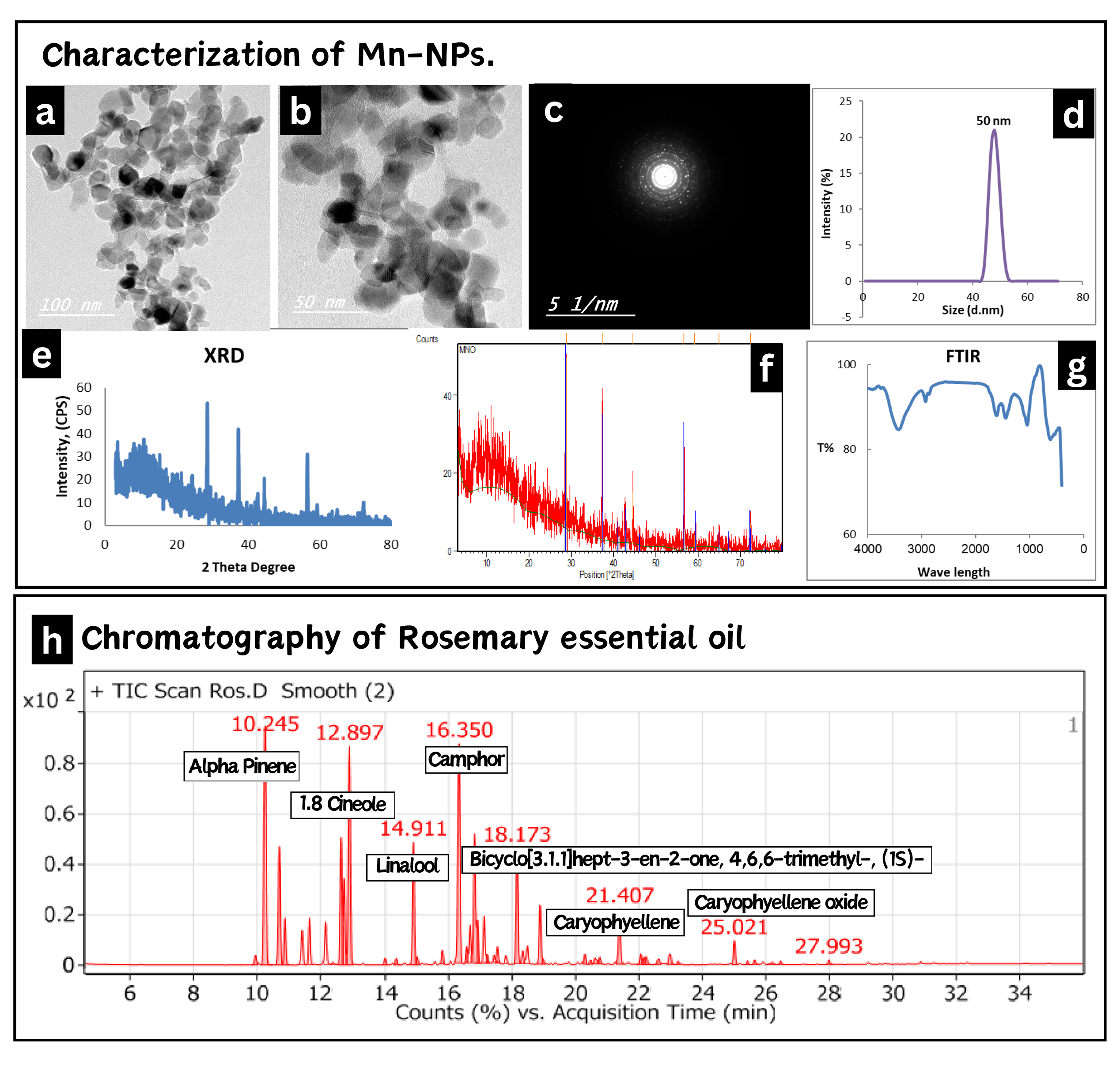

Supplement: Supplementary file 5 — Supplementary Material 5 [file 41598_2025_6345_MOESM5_ESM.png]
