# Supplementary material for: Rosemary essenitial oil counters MnO2 nanoparticle-induced fertility deficits in rats via antioxidant mechanisms and upregulation of StAR signalling
Source: Sci Rep. 2025 Jun 20;15:20201. doi: 10.1038/s41598-025-06345-7 (PMC12181236; doi:10.1038/s41598-025-06345-7)
Supplement: Supplementary file 2 — Supplementary Material 2 [file 41598_2025_6345_MOESM2_ESM.pdf]

The Institutional Animal Care and Use Committee

Vet. CU. IACUC

Letter of IACUC Protocol Approval

Vet CU 25122023884

Effects of some nanoparticles on some physiological parameters in male Sprague Dawley rats.

Department of **Physiology**; Faculty of Veterinary medic

Dear **Dr. Hager Magdy Ramadan** (Ahmed Youssef ; Asmaa Safwat ;Nadia Ahmed Taha).
The Institutional Animal Care and Use Committee (IACUC) has APPROVED the above referenced Animal Use Protocol (AUP).

Date of Approval: 25/12/2023

Date of Expiration: 24/12/2024

During this one year approval period, annual reviews are required. The IACUC staff will make every effort to send the Principal Investigator annual reminders. However, the Principal Investigator is responsible for submitting an Annual Review in advance of the annual review due dates to ensure continuing IACUC approval. It is very important that these deadlines are not missed. Failure to submit an Annual Review on time will result in in the termination of the protocol.

To continue this research beyond the three years approval period, a new protocol submission will be required. To avoid a lapse in IACUC approval, it is essential that the completed renewal protocol be submitted and approved by the IACUC prior to its expiration date.

Any activities conducted under the protocol after expiration will be in direct violation of IACUC policies. It is the responsibility of the Principal Investigator to notify the IACUC of any proposed changes regarding the work described within this protocol. The Principal Investigator agrees that no such changes will be implemented until approved by the IACUC, except where necessary to eliminate apparent immediate hazards to person(s) and/or animal(s).

Prof. Fathy F. Mohamed

Fathy F. Moh

Vet.CU.IACUC Chair

iacuc@vet.cu.edu.eg

CamScanner
